# A human pancreatic ECM hydrogel optimized for 3-D modeling of the islet microenvironment

**DOI:** 10.1038/s41598-022-11085-z

**Published:** 2022-05-03

**Authors:** Daniel M. Tremmel, Sara Dutton Sackett, Austin K. Feeney, Samantha A. Mitchell, Michael D. Schaid, Erzsebet Polyak, Peter J. Chlebeck, Sakar Gupta, Michelle E. Kimple, Luis A. Fernandez, Jon S. Odorico

**Affiliations:** 1 Division of Transplantation, Department of Surgery, School of Medicine and Public Health, University of Wisconsin-Madison, Madison, WI USA; 2 Department of Medicine, University of Wisconsin-Madison, Madison, WI USA; 3 William S. Middleton Memorial Veterans Hospital, Madison, WI USA; 4 Loyola University Medical Center, Maywood, IL USA

**Keywords:** Cell biology, Endocrine system and metabolic diseases, Biological models

## Abstract

Extracellular matrix (ECM) plays a multitude of roles, including supporting cells through structural and biochemical interactions. ECM is damaged in the process of isolating human islets for clinical transplantation and basic research. A platform in which islets can be cultured in contact with natural pancreatic ECM is desirable to better understand and support islet health, and to recapitulate the native islet environment. Our study demonstrates the derivation of a practical and durable hydrogel from decellularized human pancreas that supports human islet survival and function. Islets embedded in this hydrogel show increased glucose- and KCl-stimulated insulin secretion, and improved mitochondrial function compared to islets cultured without pancreatic matrix. In extended culture, hydrogel co-culture significantly reduced levels of apoptosis compared to suspension culture and preserved controlled glucose-responsive function. Isolated islets displayed altered endocrine and non-endocrine cell arrangement compared to in situ islets; hydrogel preserved an islet architecture more similar to that observed in situ. RNA sequencing confirmed that gene expression differences between islets cultured in suspension and hydrogel largely fell within gene ontology terms related to extracellular signaling and adhesion. Natural pancreatic ECM improves the survival and physiology of isolated human islets.

## Introduction

The extracellular matrix (ECM) is an intricate network of proteins and polysaccharides that provides structure and biological signaling to the cells residing in each tissue of the body. In 2-D cell culture, generic ECM is sometimes supplemented to support cell growth, but in 3-D cultures and engineered tissues, ECM is often tailored to the cell or tissue type^1^. Isolated human pancreatic islets are used in vitro to study the characteristic physiology and function of islet endocrine cells, for diabetes drug discovery research, and for testing new drugs for potential β cell toxicity^2,3^. Isolated islets are also used clinically as a β cell-replacement therapy for diabetes^4,5^. Significant cell death throughout the process of isolation, culture, transplantation and engraftment prevent the therapy from achieving long-term euglycemia in many patients. Non-ECM treatments that recapitulate physiological conditions in culture have recently been reported to enhance islet health and function in vitro^6,7^.

It is well established that the process of isolating islets from the pancreas, which requires the use of collagenase and neutral protease, significantly damages the islet ECM^8–10^. The lack of ECM in islets is known to induce anoikis-mediated apoptosis, and has been shown to negatively affect islet function^11,12^. Several studies have supplemented isolated islets with purified ECM molecules in culture in a variety of ways, demonstrating improved islet health and function with restored ECM contact (reviewed in Stendahl et al., 2009)^13^. Due to the complexity of the native matrisome^14^, it has been postulated that decellularized tissue may be a superior scaffold compared to artificial and incomplete ECM environments^15–17^, and that pancreas-specific ECM may have a beneficial effect on islets^18^. Furthermore, co-transplanted ECM may have a beneficial effect on the engraftment and survival of transplanted islets^19^.

Murine, porcine, and human pancreata have been decellularized in previous studies with the intention of incorporating pancreatic ECM into 3-D cell culture models, or the recellularization of an intact decellularized organ^18,20–27^. While relatively short protocols efficiently decellularize mouse and rat pancreata, much longer treatments are necessary for larger pig and human organs. Due to species-specific differences in pancreas and islet biology^28^, potential variance in ECM composition by species^13,29,30^, and structural differences in the basement membrane architecture surrounding the islets^31^, a human pancreas ECM scaffold may be more desirable for use with human cells. In previous work, we produced a hydrogel from human pancreas ECM^26^, but high lipid content of the human pancreas prevented this protocol from being applied universally to all pancreata, and the hydrogel generated required molding into tissue culture wells and were not stable enough to maintain their shape and structure independently. For practical in vitro applications and for ease of transplantation studies, a durable and pliable gel which does not lose its shape in the absence of a mold is desirable.

In this study, we have optimized the decellularization (decell) of human pancreas for improved lipid removal, which resulted in the formation of a durable human pancreas ECM hydrogel (hP-HG). The resulting gel can easily be combined with β cells or islets for in vitro culture. Human islets cultured in hP-HG exhibit a significant improvement in stimulation index as well as improved survival compared to islets maintained in suspension culture. This natural matrix provides for improved islet health, architecture and physiology and may help overcome some of the long-term culture challenges that befall isolated islets due to the loss of native ECM.

## Results

### Protocol for the decellularization and gelation of human pancreas ECM

**Figure 1:**
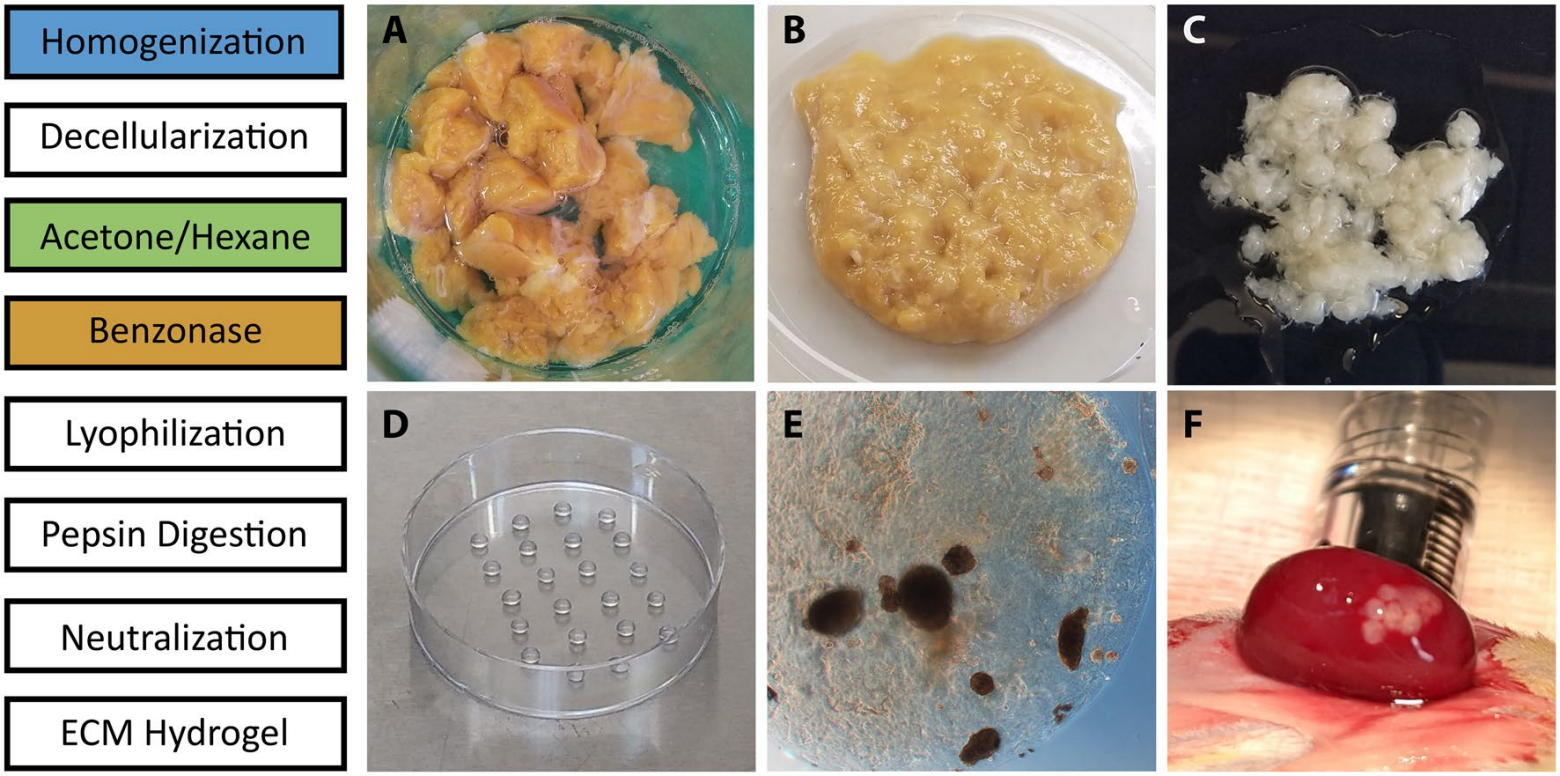
Protocol for the decellularization and gelation of human pancreas ECM. A schematic representation for the protocol to decell, digest and form a hydrogel from human pancreas ECM (left side). Images of the native tissue **(A)**, homogenized tissue **(B)**, decellularized and delipidized ECM **(C)** and multiple 5 μL hydrogel droplets in a 6 cm dish **(D)**. Human islets can be embedded in the hydrogel prior to gelation to form stable droplets for in vitro culture **(E)**; the droplets are durable enough to maintain shape and consistency throughout the transplantation process **(F)**.

Retained lipid content following decell is a barrier to robust gelation of solubilized ECM. We designed an optimized decell protocol to isolate ECM from the human pancreas with minimal lipid and DNA retention, while retaining native ECM proteins and sulfated glycosaminoglycans (sGAG). We revised our previously published protocol by adding an organic solvent wash step to enhance lipid removal, and Benzonase treatment to improve nucleic acid removal. The optimized human pancreas decell protocol resulted in isolated ECM that was capable of forming a stable hydrogel following pepsin digestion, neutralization and warming to 37 °C (Fig. 1).

### Optimized decell protocol removes lipids and DNA, resulting in an improved hydrogel

**Figure 2:**
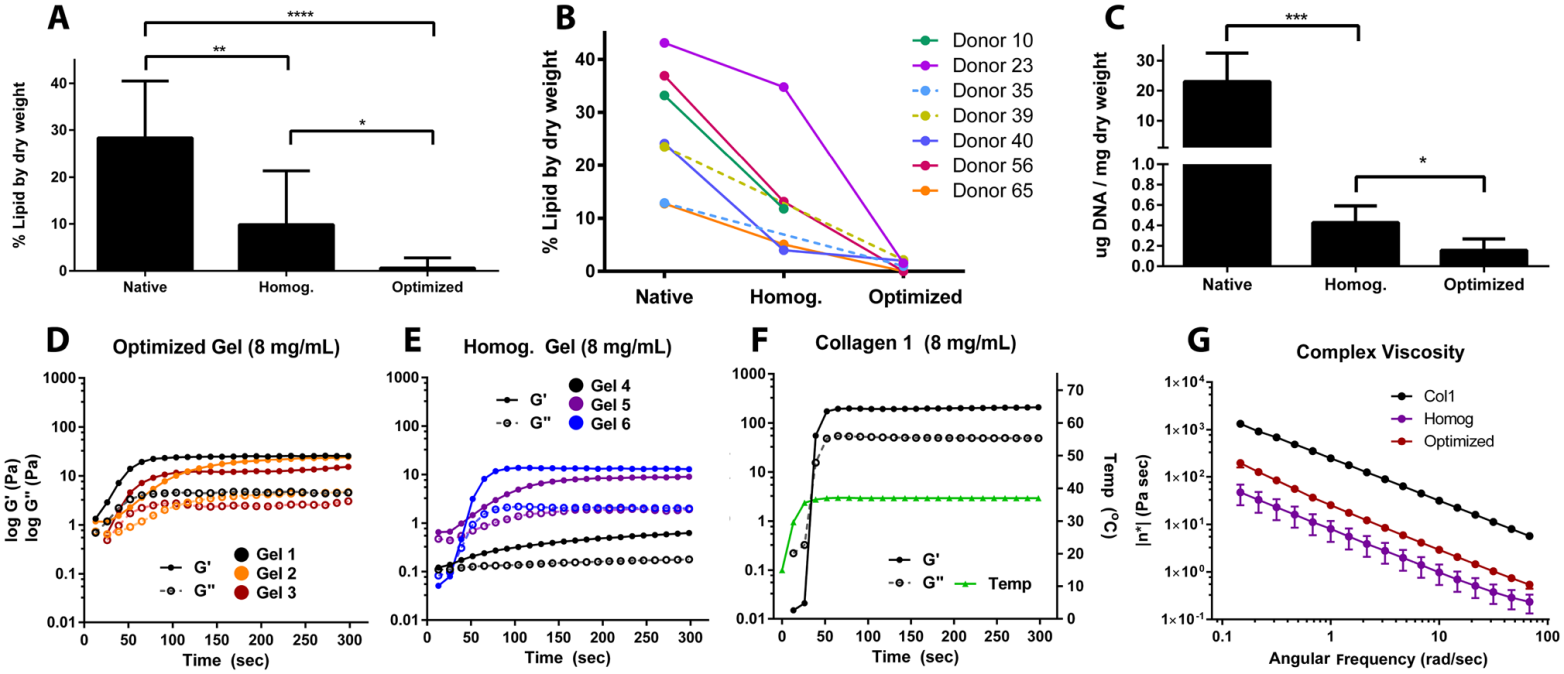
Optimized decell protocol removes lipids and DNA, resulting in an improved hydrogel. **(A,B)** Total lipid content by dry weight of the native and decellularized hP-ECM from the Homog and Optimized protocols, determined using a modified Folch method **(A)**, also displayed on a donor-by-donor basis, in which donors with higher lipid content retain significantly more lipids in the Homog protocol **(B)**. **(C)** Total DNA content of the native and decellularized hP-ECM from the Homog and Optimized protocols. **(D–F)** The storage (G’) and loss (G’’) moduli of the optimized hydrogels were less variable than the Homog hP-HG hydrogels, and compared to Col1 controls; temperature is plotted in green **(F)**. **(G)** The complex viscosity curves of the Optimized protocol are less variable than the Homog protocol, and all hP-HG gels were less firm than Col1 hydrogel of the same concentration. (*p < 0.05, **p < 0.01, ***p < 0.001, ****p < 0.0001).

The optimized protocol (“Optimized”) was compared to the previously published homogenization decell protocol (“Homog”) through the quantification of retained DNA and lipid in the isolated human pancreas ECM (hP-ECM). The lipid content derived from the optimized protocol (0.63% lipid by dry weight) was significantly lower than that of the Homog protocol (9.83% lipid by dry weight) (Fig. 2A). On an individual donor basis, the Homog protocol was less effective at removing lipids from pancreata with higher initial lipid content, while the Optimized protocol removed lipids equally well from any donor pancreas (Fig. 2B). Delipidization of lipid-laden pancreata correlated with stable hydrogel formation. The DNA content following implementation of the Optimized protocol (0.16 ± 0.06) was significantly lower than the Homog protocol (0.43 ± 0.07) (Fig. 2C). The addition of hexane, acetone and Benzonase treatment did not reduce the sGAG content of the hP-ECM compared to the Homog protocol (Supplemental Fig. 1A). Furthermore, the decelled material retained many of the structural ECM proteins found in the human pancreas (Supplemental Fig. 1B).

When hP-ECM was pepsin-digested to form a hydrogel, the Optimized protocol resulted in gels with more consistent rheologic properties compared to the Homog protocol. This was evident through more uniform clustering of the sigmoidal storage and loss moduli curves (G’ and G”) in the Optimized hydrogel (Fig. 2D), compared to the broader range of these curves from the Homog protocol (Fig. 2E). Purified collagen 1 (Col1) hydrogel and temperature plots are included for reference (Fig. 2F). Additionally, the curve of complex viscosity vs. angular frequency was shifted higher for the Optimized compared to the Homog protocol (Fig. 2G), with an increased Young’s Modulus (Supplemental Fig. 2C), indicating that the Optimized protocol produced a firmer gel with more consistent and reproducible rheological results than the previous protocol. These rheological properties corresponded with rigid gels that held shape better when pipetted into droplets (Supplemental Fig. 2A,B), similar to Col1 gels. The resulting gels can easily be picked up with forceps (Supplemental Video 1), enabling use in cell culture, functional assays, and transplantation (Supplemental Fig. 2D,E). Similar to other ECM hydrogels, the acellular hP-HG scaffold is capable of gelation in vivo and was found to be highly compatible with minimal immune infiltration when tested in a humanized mouse model (Supplemental Fig. 3).

### hP-HG co-culture improves islet function after 2 days of culture

**Figure 3:**
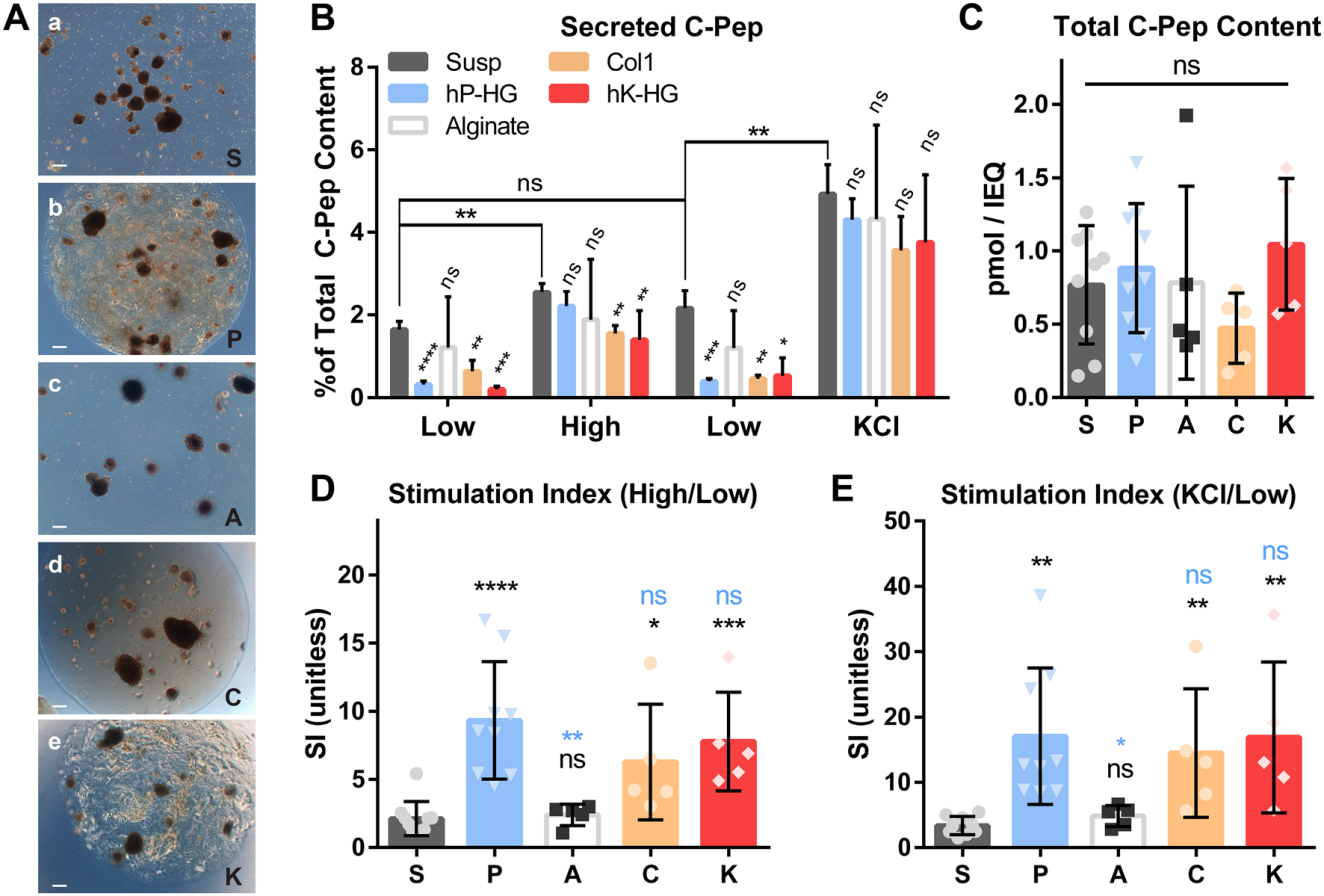
hP-HG co-culture improves islet function after 2 days of culture. **(A)** Representative phase contrast images of islets cultured in suspension (“S”) (a), hP-HG (“P”) (b), alginate (“A”) (c), Col1 (“C”) (d), or hK-HG (“K”) (e) were assessed for function on Day 2 of culture (scale = 200 microns). **(B)** A static glucose stimulated insulin secretion (GSIS) assay was performed using sequential low glucose (2.8 mM, “Low”), high glucose (28 mM, “High”), a return to low glucose, followed by low glucose + KCl (30 mM KCl, “KCl”); basal and stimulated C-peptide (C-pep) secreted during the static GSIS with human islets are shown as a percentage of total C-pep content. Statistics indicated above each bar are relative to the “S” control for the same treatment. **(C)** Total C-pep content of islets undergoing the indicated treatments. Stimulation index **(D**, high/low glucose) (**E**, KCl/low glucose) of human islets cultured in all five conditions, determined by static GSIS. Statistical comparisons indicated in black are relative to “S”, and indicated in blue are relative to “P”. (S,P: n = 9; A,C,K: n = 5 islet donors) (*ns* not significant, *p < 0.05, **p < 0.01, ***p < 0.001, ****p < 0.0001).

The generation of a stable hydrogel using the Optimized decell protocol enabled 3-D studies combining hydrogel and human islets which were not possible with the more fragile Homog protocol. The Optimized protocol was also employed for use in kidney decellularization to generate a human kidney ECM hydrogel (hK-HG) as a control for initial experiments. Isolated islets were cultured in suspension (“S”) (Fig. 3A-a), embedded in hP-HG (“P”) (Fig. 3A-b), alginate (“A”) (Fig. 3A-c), purified Col1 (”C”) (Fig. 3A-d) and in hK-HG (“K”) (Fig. 3A-e) for 2 days. Alginate was included as a control because this material is commonly used to embed and transplant islets as a micro-encapsulation strategy^32–34^; it also provides a non-ECM-based control hydrogel environment for our study. Purified Col1 was used as a non-pancreas specific, simple ECM control, and hK-HG was included as a non-pancreas specific, complex ECM control.

After 2 days of culture in each condition, a static glucose stimulated insulin secretion (GSIS) assay was performed to assess islet function. Islets in each treatment group were sequentially exposed to low glucose (2.8 mM), high glucose (28 mM), low glucose, and KCl (30 mM); secreted C-pep is plotted as a percentage of the total C-pep content. Alginate encapsulation did not affect islet secretion under any of the four conditions compared to suspension culture. All ECM-treated groups (P,C,K) displayed a reduced basal insulin secretion compared to suspension. Furthermore, islets embedded in hK-HG, and Col1 had significantly reduced stimulated C-pep secretion in high glucose compared to S,P, or A. Islets in hP-HG, however did not have significantly different stimulated secretion in either high glucose or KCl compared to suspension. Therefore, islets in hP-HG culture had a significantly higher stimulation index (mean SI = 9.34) in static GSIS compared to islets in suspension (SI = 2.12) and alginate (SI = 2.40), and improved compared to Col1 (SI = 6.28) and hK-HG (SI = 7.78) (Fig. 3D). The stimulation from low glucose to KCl followed the same trend among the treatment groups (Fig. 3E). Total C-pep content was not significantly different among the five treatments (Fig. 3C). All groups reverted to a lower C-pep secretion in the second low glucose step, indicating that hydrogel does not impair the return to basal secretion following stimulation (Fig. 3B). Because Col1 and hK-HG had significantly reduced basal and stimulated insulin secretion compared to suspension and hP-HG cultures, these treatments were considered less desirable for islet function, despite having good stimulation. An alternative representation of these data relative to the basal C-pep secretion in suspension culture normalized to each individual islet donor are included in Supp. Fig. 4G.

### hP-HG culture enables proper dynamic function and enhances maximum respiration

**Figure 4:**
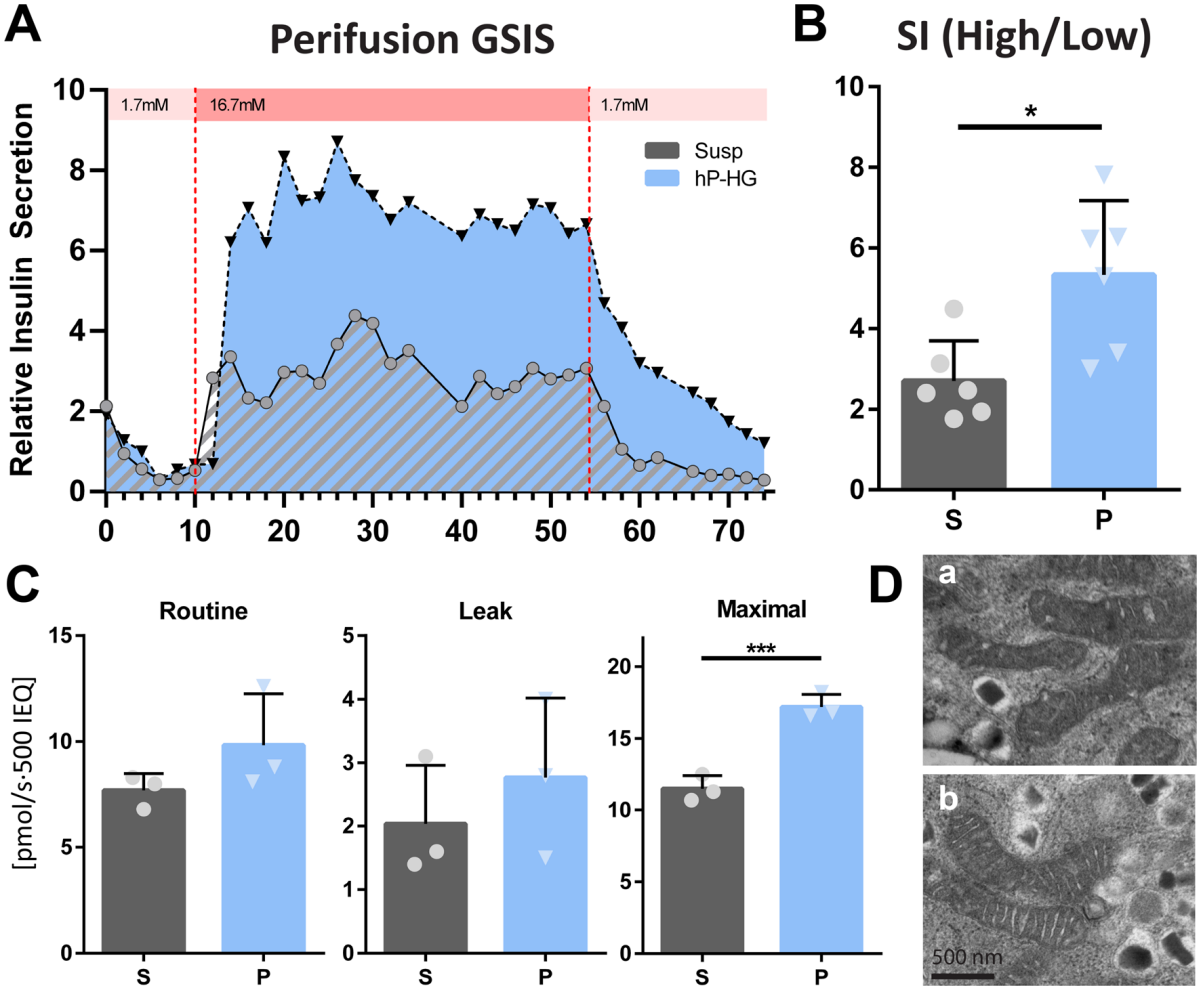
hP-HG culture enables proper dynamic function and enhances maximum respiration. **(A)** Perifusion GSIS was performed on human islets after 2 days of culture in either suspension (S) or embedded in hP-HG (P), from low glucose (1.7 mM) to high glucose (16.7 mM) and back to low glucose; representative plots shown were generated from 2 technical replicates per treatment, from one islet donor. Insulin secretion is normalized to first low glucose response (unitless). One representative donor is shown, data from additional donors can be found in Supplemental Fig. 4D,E. **(B)** Average stimulation index (SI, high/low glucose) is reported, N = 3 islet donors. **(C)** Islets were assessed for mitochondrial respiration after 2 days of culture in either suspension (S) or hP-HG (P); Routine (normal media), Leak (Complex V inhibition), and Maximal (uncoupled) respiration were measured. **(D)** β cell mitochondrial pathology in suspension (a) and hP-HG (b) culture was assessed with transmission electron microscopy. N = 3 islet donors. Scale = 500 nm. (*ns* not significant, *p < 0.05, ***p < 0.001).

To further characterize the effect hP-HG had on islet health and function, additional studies were performed comparing only suspension (S) and hP-HG (P). To assess the dynamic insulin secretion profile of the islets in the hydrogel, perifusion GSIS was performed on human islets after 2 days of culture either in suspension or in hP-HG (Fig. 4A) (Supplemental Fig. 4E,F). The perifusion assay revealed that islets in hP-HG had only a minor delay (1–2 min) in response to increased glucose concentration and properly suppressed insulin secretion upon return to low glucose. As with the static GSIS, the stimulation index was higher in hP-HG cultured islets (Fig. 4B). Among the islet donors assayed in static or perifusion GSIS, all but one islet prep had an increased SI when cultured in hP-HG compared to suspension (Supplemental Fig. 4H).

As further confirmation of the protective role of hP-HG culture on β cell function, INS1 832/13 rat insulinoma cells were aggregated as pseudoislets and embedded in hP-HG droplets for 2 days of culture. INS1 cells showed a significantly improved static GSIS stimulation index from low to high glucose, (Supplemental Fig. 4A), an increased SI with KCl (Supplemental Fig. 4B), as well as an increased total C-pep content compared to suspension clusters (Supplemental Fig. 4C). Like human islets, the increase in SI in hP-HG co-culture was due to significantly lower insulin secretion under low glucose conditions, while the insulin secreted in high glucose was not significantly different (Supplemental Fig. 4D).

To assess whether differences in mitochondrial function may underlie glucose-responsive insulin secretion between islets cultured in hP-HG and suspension, high resolution respirometry was performed. We tested the physiological function of mitochondria by measuring the mitochondrial oxidative phosphorylation (OXPHOS) by detecting oxygen consumption and calculating the rate of oxygen consumption at different respiratory stages in intact cells. Human islets cultured in hP-HG had 27.7% higher, but not significantly different, levels of basal mitochondrial respiration compared to suspension culture (Fig. 4C, Routine), and similarly a 36.5% increase following inhibition of ATP synthase (Complex V) to stop ATP generation (Fig. 4C, Leak). However, after uncoupling the phosphorylation system with FCCP, mimicking a physiological energy demand, islets cultured in hP-HG had a 44.1% significantly increased maximal respiratory capacity of the mitochondria compared to those cultured in suspension (Fig. 4C, Maximal). The change in maximal mitochondrial respiration was not found to be accompanied by a measurable change in mitochondria size or shape, as visualized through TEM (Fig. 4D, Supp Fig. 5).

### hP-HG co-culture improves islet survival and function in extended culture

**Figure 5:**
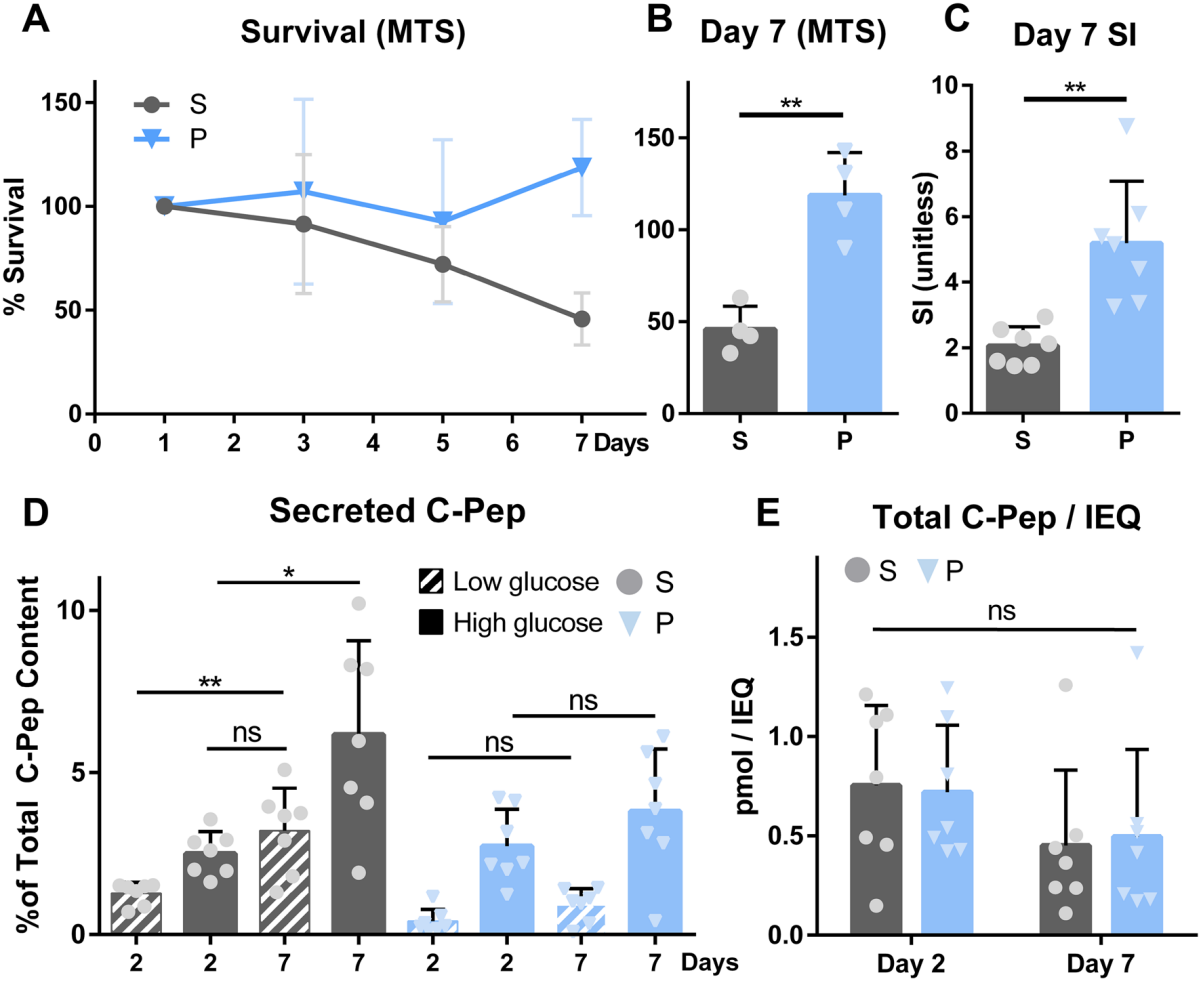
hP-HG co-culture improves islet survival and function in extended culture. (**A,B)** Metabolic activity of islets cultured in suspension (S) or hP-HG (P) over a 7 day period was assessed by MTS assay, reflecting islet survival over time (presented as a percentage of the Day 1 value). No significant difference in survival was apparent on day 3 or 5 **(A)**, but a significant difference between S and P was found on day 7 **(B)**. N = 4 islet donors per treatment. **(C–E)** Islet function changed over the 7 day period, as assessed through static GSIS. Islets maintained a significantly higher stimulation index (ratio of C-pep secreted under high/low glucose) at day 7 **(C)**. The percentage of total C-pep content secreted under low and high glucose is plotted for day 2 and day 7 under both suspension and hP-HG culture conditions **(D)**. Total C-pep content was not significantly different among the two treatments at the two time points **(E)**. N = 7 islet donors. (*ns* not significant, *p < 0.05, **p < 0.01).

To assess the effect of hP-HG on islet survival in extended culture, islets were kept in suspension or hP-HG for 7 days. Islets were counted and plated on “day 0” and measured on days 1, 3, 5, and 7 thereafter using an MTS assay to assess survival. The same number of starting islets were used at all time points, so islet attrition is reflected in a reduction in MTS response as time progresses. As expected, islets in suspension displayed a steady decline in MTS activity over the 7-day period, suggesting cell death. Islets cultured in hP-HG, however, had stable metabolic activity in the first few days, and somewhat greater than 100% metabolic activity by day 7 (Fig. 5A). The MTS measurements on days 3 and 5 were not significantly different, however by day 7 there was significantly higher MTS response in the hydrogel-cultured compared to suspension-cultured islets (Fig. 5B). Islets cultured in hP-HG had noticeable outgrowth into the gel by day 7 (Supplemental Fig. 6), a potential reason for the increased metabolic activity. Islet function was also assessed on days 2 and 7 through static GSIS. hP-HG co-cultured islets maintained a significantly higher stimulation index from low to high glucose at day 7 (Fig. 5C), and displayed stable secretion levels after one week of culture in both low and high glucose (Fig. 5D). Islets cultured in suspension, however, secreted a significantly higher amount of their stored C-pep on day 7 compared to day 2, with significant increases in percent secretion under both low and high glucose. Of note, under suspension conditions, the percentage of C-pep secreted in low glucose (basal) at day 7 (average = 3.2% of total C-pep) was consistently equal to or higher than that in high glucose (stimulated) at day 2 (average = 2.5% of total C-pep). This level of leaky insulin secretion after a week of culture is indicative of islet dysfunction, and was not observed in hP-HG islet cultures. Total C-pep per IEQ was not significantly different among the two treatments at the two time points (Fig. 5E).

### Apoptosis rates and islet architecture are altered in suspension culture and partially preserved in hP-HG

**Figure 6:**
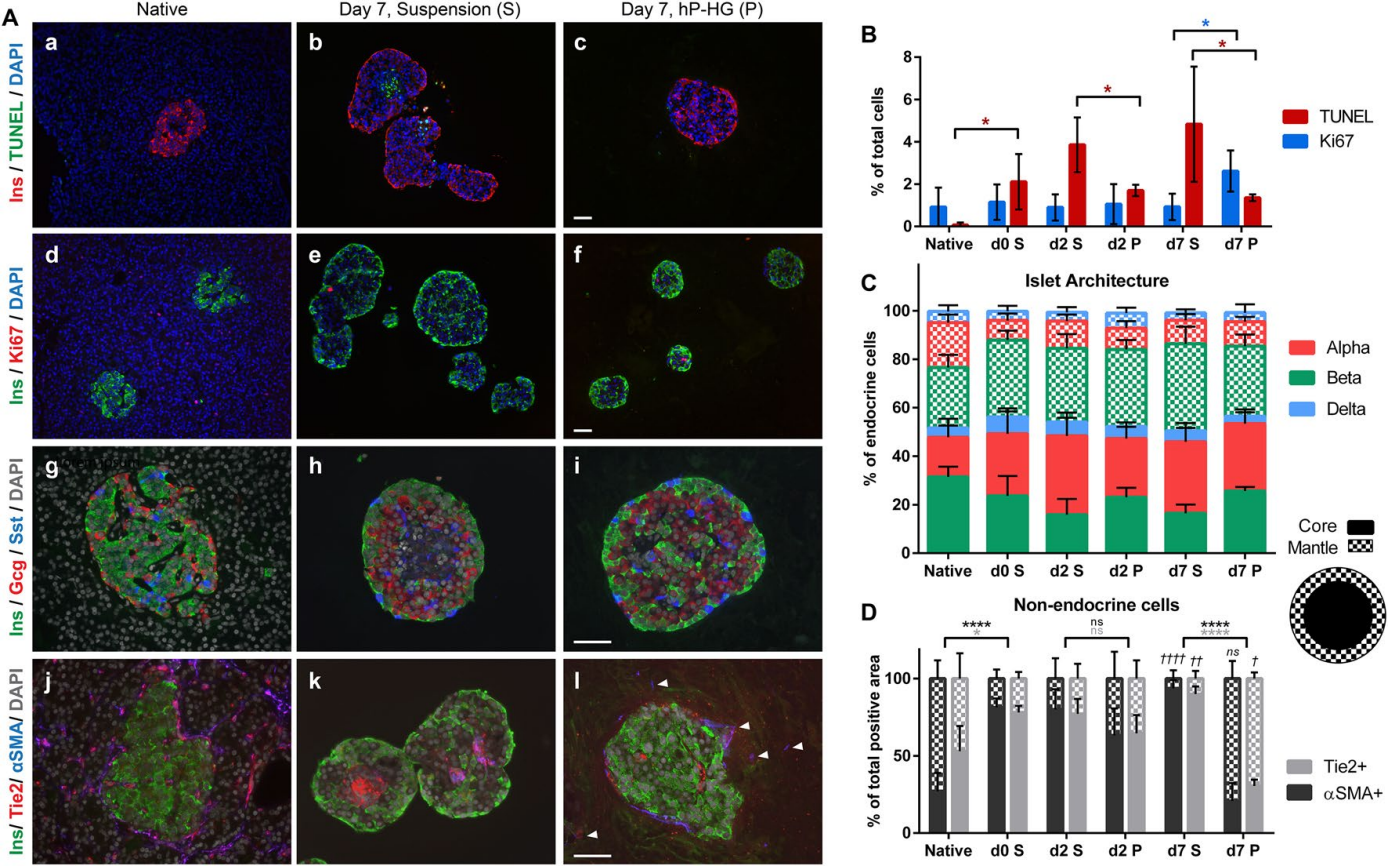
Apoptosis rates and islet architecture are altered in suspension culture and partially preserved in hP-HG. **(A)** Immunofluorescent staining images for native (in situ) islets, and islets cultured in suspension or hP-HG for 7 days. Islets are stained for TUNEL/Insulin (a–c), Ki67/Insulin (d–f), insulin/glucagon/somatostatin (g–i), and insulin/Tie2/αSMA (j–l). White arrowheads indicate projections of Tie2^+^ and αSMA^+^ cells into the hydrogel. All scale bars = 50 microns. **(B)** Quantification of cells positive for Ki67 (blue bars, significance indicated by blue stars) and TUNEL (red bars, significance indicated by red stars) in native islets, and on day (d) 0, 2 and 7 in suspension (S) and hP-HG (P) culture. N = 4 islet donors. **(C)** Assessment of islet architecture by counting α (Gcg^+^), β (Ins^+^), and δ (Sst^+^) cells in the islet mantle (checkered) and core (solid). Data is represented as a percentage of total endocrine cells. N = 5 islet donors. (Statistical analyses presented in Supplemental Fig. 7D,E.) **(D)** Localization of positive staining for Tie2 (light gray) and αSMA (dark gray) in the islet mantle (checkered) and core (solid). N = 5 islet donors. p-values depicted in italics and with the † symbol are relative to the native tissue. (*ns* not significant, */*†*p < 0.05, **/*††*p < 0.01, ****/*††††*p < 0.0001).

Immunofluorescent (IF) staining was performed to investigate cell health in suspension and hP-HG culture. TUNEL staining was used to assess apoptosis in the islets over time (Fig. 6A,a–c). Consistent with the MTS survival curves, there were significantly higher rates of apoptosis in islets in suspension compared to islets embedded in hP-HG after 7 days of culture (Fig. 6B, red bars). Ki67 staining was assessed to determine relative levels of cell proliferation under the two conditions (Fig. 6A,d–f). Rates of proliferation were low in all samples, but significantly higher in hydrogel-embedded islets after 7 days of culture (Fig. 6B, blue bars). Ki67^+^ staining, although only quantified within islets, does not appear to co-localize with insulin^+^ cells.

Markers for each major endocrine cell type were visualized with IF staining, revealing a change in endocrine architecture following isolation (Fig. 6A,g–I; Supplemental Fig. 7F). Islet sections were quantified for numbers of each endocrine cell type (α, β, δ), interactions of each cell with neighboring cells (α–α, α–β, etc.) and localization of each cell at the mantle or core of the islets. Total cell type composition (Supplemental Fig. 7A), cell–cell interactions (Supplemental Fig. 7B,C), and the ratio of non-endocrine cells (Supplemental Fig. 7G) were not found to be significantly different over 7 days in culture, or in suspension versus hP-HG treatment. In contrast, the mantle-core arrangement of endocrine cells was significantly altered in isolated islets compared to native islets (Fig. 6C). After isolation, islet β cells were more likely to localize to the mantle than the core, while the native islets consistently have about equal distribution of the three endocrine cell types throughout the entire islet, as previously described^35^. By day 7, the distribution of endocrine cell populations in the mantle and core was partially preserved in hP-HG, with a significantly higher fraction of β cells remaining in the center of the islets (47%), compared to suspension (31%) (Supplemental Fig. 7D). Despite a significant difference in β cell localization between suspension and hydrogel-embedded islets, α cells localized in the core at a very high rate (75%) on day 0 of culture, and did not significantly change over the course of the experiment regardless of treatment (Supplemental Fig. 7E, representative images in Supplemental Fig. 7F, Fig. 6A).

Interestingly, the localization of resident non-endocrine cells in the isolated islets also changed dramatically following isolation (Fig. 6A,j–l; Supplemental Fig. 7H), with the majority of TEK tyrosine kinase (Tie2)-positive cells and alpha smooth muscle actin (αSMA)-positive cells localizing toward the center of the islets post-isolation, and throughout culture in suspension. After 7 days in hP-HG, the majority of the Tie2^+^ and αSMA^+^ cells localized at the islet mantle and expanded into the gel. The arrangement of these cells in hP-HG culture was significantly more similar to that of the native in situ islets than in suspension culture (Fig. 6D).

### Extracellular signaling-related pathways are significantly influenced by hP-HG culture

To identify whether the biological mechanisms through which hP-HG co-culture improves islet survival and function are related to ECM signaling, we performed bulk RNA sequencing. Following 2 days of culture in either suspension or hP-HG, islets were lysed to collect RNA, and assessed for differentially expressed genes (DEGs). Out of 17,975 total transcripts identified, 1633 were found to be significantly different between the suspension and hP-HG cultures. Of these, 1078 genes were expressed at higher levels in hP-HG culture and 555 genes were expressed at higher levels in suspension culture. Gene Ontology (GO) analysis of the DEGs resulted in the identification of 30 GO terms in the Molecular Function (MF) aspect, 191 GO terms in the in the Biological Processes (BP) aspect, and 27 GO terms in the Cellular Component (CC) aspect (Supplement Fig. 8A). The most significant terms in each aspect related to the extracellular space, cell adhesion, receptor signaling, vesicles/exocytosis, and cytokines/inflammation (Supplement Fig. 8B). Gene expression for key endocrine markers, including insulin, were not significantly different between the two groups (Supplemental Table 3).

## Discussion

Our study aimed to rebuild the 3-D ECM microenvironment of the human pancreas as a hydrogel that can easily be integrated into in vitro culture and is compatible with transplantation. Due to differences in cell behavior between 2-D and 3-D environments, a 3-D islet microenvironment model could be useful for creating better microphysiological islet systems^36^; such may be useful for drug discovery and toxicity testing^37^. We have demonstrated a new protocol for decellularization that removes lipids and DNA from human pancreatic tissue and forms a more stable gel derived from pancreata with substantial lipid content. The decell and gelation process retains collagens well, but there is some reduction in glycoproteins and other ECM components, including a reduction in sGAG content, consistent with previous publications^23,26,27^. This enrichment for collagens and reduction of other ECM components has also been shown in several other pancreas decell studies^18,21,22,26,27,38,39^. Recent work characterizing the human pancreatic matrisome indicates that collagens are the most abundant ECM proteins in the pancreas, and are relatively evenly distributed between the acinar and islet compartments^14^, therefore a whole-pancreas hydrogel consisting of mainly pancreatic collagens may be representative of the native islet ECM environment. This is also an efficient method of constructing a hydrogel made up of these basic ECM components, which individually in purified form are very expensive.

Islets undergo significant injury throughout the process of isolation from the pancreas, and in particular the ECM is heavily damaged leading to anoikis-mediated apoptosis. We hypothesized that replacing the ECM component of the islet microenvironment would help improve islet survival and function in vitro. We found that INS1 cells and primary human islets exhibited positive changes in GSIS when embedded in hP-HG. Consistently, the insulin secreted under basal (low glucose) conditions was significantly reduced compared to suspension cultures, while stimulated insulin secretion was unchanged, resulting in an increased stimulation index. Interestingly, a similar trend of increased stimulation index due to reduced basal insulin secretion was recently reported with human islets entrained to daily feeding/fasting rhythms using various stimuli (glucose, arginine, forskolin, and insulin) in culture^6^. Together, this suggests that an improved stimulation index and reduced basal insulin secretion may better reflect the function of in situ islets compared to isolated islets in suspension. Similarly, Singh et al. recently found that the culture of stem cell-derived beta cells with various ECM molecules reduced the basal insulin secretion, and improved GSIS^40^. Several other studies have presented an improved stimulation index in islets cultured with ECM without reporting the raw insulin secretion values under low and high glucose treatment^41–43^, which makes comparison of our study to others unclear. However, other studies have demonstrated that ECM treatment promotes an increase in the stimulated insulin secretion without affecting the basal level^18,44^. These disparities in response to glucose may be due to differences in ECM composition, co-culture method, or GSIS assays performed using different concentrations of low and high glucose solutions, and warrant further investigation.

After 7 days of culture, islets in suspension secreted more insulin under basal conditions compared to day 2, so much so that the basal insulin secretion on day 7 was higher than the stimulated insulin secretion on day 2 of culture. This trend was not observed in islets cultured in hP-HG, suggesting better maintenance of controlled function throughout the culture period. Islets in hP-HG also had significantly improved survival over 7 days of culture compared to suspension, as assessed through an MTS assay. Immunofluorescent staining corroborated these findings, indicating that islets in suspension have greater levels of apoptosis (TUNEL^+^ cells) and reduced levels of proliferation (Ki67^+^ cells) compared to islets cultured in hP-HG. Furthermore, on day 2 of culture, islets in hP-HG had higher maximal mitochondrial function compared to suspension-cultured islets. Mitochondria are crucial in the generation of ATP in response to glucose, affecting both the closure of ATP-sensitive K^+^ channels and Ca^2+^ influx^45–47^, which are necessary for insulin secretion. The mitochondria of islets cultured in hP-HG are better equipped to respond to a metabolic challenge, possibly due to maintenance of healthier mitochondrial dynamics compared to islets cultured without hP-HG.

ECM scaffolds have both mechanical and biochemical roles in the cell environment. After two days of culture, islet function was assessed in five different environments: suspension (S), hP-HG (P), alginate (A), Col1 (C) and hK-HG (K), with a trend in stimulation from low to high glucose: SI_S_ < SI_A_ < SI_C_ < SI_K_ < SI_P_. This suggests that the mechanical environment of a hydrogel (P, A, C, K) compared to suspension culture may contribute to better islet function. It is also possible that ECM-signaling (P, C, K) may contribute more to islet function than the mechanical environment alone (A), and potentially reveals the importance of a complex mixture of natural ECM proteins (P, K), compared to a singular ECM component like Col1 (C). Finally, the pancreas-specific ECM (P) outperformed the kidney-specific ECM (K) in regard stimulated C-peptide secretion levels, suggesting that the ECM composition of the pancreas itself may have a beneficial effect on islet health and function. Importantly, the generation of these scaffolds for our study was focused on controlling for ECM protein content, and not matching the gel stiffness which has previously been shown itself to have an effect on β cell function^34,48^. However, a recent study by Enck et al. has also elucidated that ECM has a profound effect on islet function independent of gel stiffness^34^. The RNA-seq GO analyses do indicate that hP-HG co-culture activates significant enrichment in ECM-signaling and adherence-related ontologies, suggesting that islet-ECM signaling helps maintain endocrine cell health.

Our study identified that islet architecture is altered in culture compared to in situ. As has previously been observed, a higher fraction of β cells are found at the periphery of the islet in suspension culture compared to in the native pancreas^49,50^. In hP-HG co-culture, β cell mantle-core arrangement is more similar to native islets, although the α cells are significantly more enriched in the core than in situ. We find that islets in situ have a roughly equal distribution of α, β, and δ cells between the core and mantle; this is consistent with recent conclusions that human islets do not have a mantle-core arrangement or subunit domains similar to rodents^35^. Islets embedded in hP-HG on day 0 preserve endocrine architecture on days 2 and 7 that is insignificantly different than freshly isolated (day 0) islets; this indicates that in hP-HG, islet architecture is preserved over time relative to the day islets were embedded. Islets in suspension, however, continue to change endocrine architecture over the 7 day period. This observation is of consequence, because islet architecture is thought to affect cross-talk within the endocrine cell populations, and influence islet function^51–53^. Interestingly, resident non-endocrine cells, such as endothelial cells (Tie2^+^), vascular mural cells (pericytes, smooth muscle cells; αSMA^+^) or fibroblasts (αSMA^+^), display an even more dramatic rearrangement following isolation. Some human islets are surrounded by a capsule of αSMA^+^ cells in situ (Fig. 6A-j) and the arrangement of these cells in islets cultured in hP-HG significantly resembles this “capsule” morphology (Fig. 6A–l), while the αSMA^+^ and Tie2^+^ cells in suspension-cultured islets appear to form abnormal internal nodules (Fig. 6A-i). The mechanism through which the cells rearrange could reflect migration of the different cell types in a balance between cell-cell adhesion and cell-ECM adhesion, as has been described in other culture systems^54^, but the cellular complexity of isolated islets and affinities for each cell type with one another has not been well established. Importantly, in the present study, only the outer perimeter of the islets is in contact with the ECM hydrogel. Further studies are necessary to explore the potential mechanisms of islet cell arrangement.

Islet transplantation into the liver through the portal vein has been associated with significant islet death following transplantation^55^. Alternative strategies have been suggested for islet and stem cell-derived islet-like cluster (SC-ILC) transplantation, including transplantation into the omentum, a vascularized subcutaneous space, or within a device that can protect the cells from immune rejection^56^. A re-evaluation of transplant site and strategy invites the opportunity for the inclusion of ECM into islet culture and transplantation, particularly to support islet health during the period prior to vascularization and ECM remodeling. A recent study utilizing a collagen-based “islet viability matrix” has demonstrated that indeed, subcutaneous co-transplantation of islets with the matrix improves engraftment, function and provides immune protection^19^. Due to the conserved nature of ECM proteins, decelled ECM is hypoimmunogenic^16,26,57^ and is therefore compatible with transplantation. A first-in-human clinical trial using decelled ECM hydrogel to treat myocardial infarction has already been completed without serious adverse effects related to the hydrogel^58^. Combined, these results strongly suggest that the inclusion of ECM into islet culture and transplantation could improve the engraftment, survival, and function of the islets; future studies utilizing these materials in transplantation are underway.

Human pancreatic hydrogel may also be useful for accelerating, enhancing, or stabilizing the maturation of SC-ILCs. Due to limited availability of primary donor islets for clinical treatment, efforts have been made to differentiate SC-ILCs with the intention of transplanting functional cells to cure diabetes. Despite significant strides in the field of β cell differentiation, deficits in the maturation and function of the SC-ILCs still exist, including efficiency of β cell yield, total insulin content and secretion, and gene expression for markers of islet maturation^59^. It is thought that ECM signaling may play beneficial roles toward the determination of cell fate, and therefore may be valuable in SC-ILC differentiation^40,60,61^. Overall, hP-HG could provide a substrate for improved in vitro islet and SC-ILC culture, a material to support transplanted islets and SC-ILCs, and a scaffold for in vitro 3-D modeling of human pancreas and islet development.

The combination of islets with hP-HG as a liquid prior to gelation allows for alteration of the platform, in which many other components could be added. This could include supplemented ECM components, such as GAGs or glycoproteins that are reduced in abundance through the decellularization and digestion process. Decelled hP-ECM is composed of structural ECM proteins from the whole pancreas, which is mainly exocrine tissue; hP-HG could therefore serve as a base scaffold to which more islet-enriched ECM components could be added for future studies. It would also facilitate the inclusion of other cell types in islet culture, such as endothelial cells, neurons or immune cells, all of which have important roles in islet health and function. In current culture systems, non-endocrine cells have been combined with islet endocrine cells following single cell dispersion which does not recapitulate the in situ arrangement and structures of the various cell types^62–64^. Finally, future studies using hP-HG could incorporate oxygen generating materials, drug carriers, or immune-modulatory particles, which could all be used to support and protect islets in culture or in vivo. An added feature is that the droplets of gel can be picked up, transferred and transplanted more easily than individual islets.

## Conclusion

We have generated a hydrogel from human pancreas ECM that is easy to combine with human islets in culture and also utilizes material from discarded organs. The hydrogel co-culture system improves the survival and function of human islets after extended culture and reduces the level of apoptosis. As early as 2 days in hP-HG co-culture, islets display an improved GSIS stimulation index through a reduction in basal insulin secretion, as well as improved maximal mitochondrial function. The scaffold appears to better preserve islet architecture during culture and stimulates gene expression changes through ECM- and adhesion-mediated pathways. This islet culture platform mimics the native pancreatic niche and may provide a mechanism for better modeling islet biology and physiology in vitro.

## Methods

### Tissue procurement and ethics

Adult human pancreata (n = 10, age 21–61 years) and kidneys were obtained through the University of Wisconsin Organ and Tissue Donation with informed consent obtained for research from next of kin and authorization by the University of Wisconsin-Madison Health Sciences Institutional Review Board (IRB granted an exempt from protocol approval for studies on postnatal tissue because research on deceased donors is not considered human subjects research). IRB oversight of the project is not required because it does not involve human subjects as recognized by 45 CFR 46.102(f), which defines a “human subject” as “a living individual about whom an investigator (whether professional or student) conducting research obtains (1) data through intervention or interaction with the individual, or (2) identifiable private information.” Research was performed in accordance with federal and state law and the relevant institutional ethical committee guidelines and regulations. No organs or tissues were procured from prisoners. A list of donors used in this study and demographic data are included in Supplemental Table 1.

### Decellularization and hydrogel formation

Pancreata were trimmed of extraparenchymal fat, sectioned into 1 cm^3^ pieces, flash frozen and stored at −80 °C. For decellularization, pieces were thawed, rinsed with 1 × PBS, rinsed with water and homogenized in water until broken up. The homogenate was centrifuged (4300 x g, 5 min), floating fat removed, and supernatant discarded; the pellet was washed and centrifuged again (4300 x g, 5 min). The pellet was resuspended into 2.5 mM sodium deoxycholate/PBS and incubated, with shaking, for 3 h at room temperature (RT). After this time, the homogenate was strained over a sieve (MilliporeSigma, St. Louis, MO); all collected material was placed into fresh 2.5 mM sodium deoxycholate/PBS and incubated for an additional 15 h (RT, shaker). The ECM was strained, rinsed with water and washed in 1 × PBS supplemented with Pen/Strep for 24 h. Further lipid removal was achieved with additional steps modified from Soffer-Tsur et al.^65^. The ECM from above was strained and dehydrated with 70% ethanol for 30 min, followed by 3 washes with 100% ethanol for 30 min each. The matrix was washed with acetone 3 times, for 30 min each. The dehydrated ECM was washed with 40:60 (v:v) acetone:hexane for 24 h, with changes into fresh solution approximately every 8 h. The matrix was washed with 100% ethanol for 30 min and rehydrated with 70% ethanol overnight, followed by two washes with 1 × PBS supplemented with Pen/Strep for 24 h each. To remove nucleic acids, the ECM was treated with Benzonase (MilliporeSigma, St. Louis, MO) (500 mU/mL in 50 mM Tris, 1 mM MgCl_2_, 0.1% BSA, pH 8.0) for 18 h at 37 °C, washed with 50 mM Tris for 2 h, followed by two 24-h washes with sterile water. The resulting decellularized pancreatic ECM was lyophilized and stored at −80 °C.

Homogenization decellularization (Homog) without acetone:hexane and Benzonase treatment was performed for comparison, as previously described in Sackett and Tremmel et al.^26^.

The lyophilized ECM from each decell protocol was pepsin digested for hydrogel formation as previously described^26,66^. If necessary, the ECM was homogenized after a day in the pepsin solution to facilitate digestion. Collagen controls were prepared from rat-tail collagen type I (Corning, Corning, NY).

Human kidneys were processed in a similar timeline and manner as human pancreas, and the protocol for decellularization and hydrogel formation was the same as the Optimized protocol for pancreas.

### Lipid, DNA and GAG content

Lyophilized material was weighed to record the tissue dry weight. Lipids were extracted from each sample using a modified Folch method as previously described^26,67^. The lipid phase was dried and weighed to measure the lipid content as a percentage of the initial dry weight.

The delipidized tissue was used for DNA and GAG content analysis. Weighed and lyophilized ECM was digested with papain for 18 h at 65 °C prior to the assays. Quantification of DNA was assessed using the Quant-iT™ PicoGreen^®^ dsDNA Assay (Life Technologies, Carlsbad, CA), following manufacturer’s protocol.

### Rheology

To compare the rheologic properties of the optimized hydrogel to the previously published hydrogel, a TA Instruments AR-G2 rheometer was used. A 40 mm parallel plate geometry was used with a 500 micron gap distance; a Peltier unit was used to control temperature. First, a time sweep was performed over 10 min; the temperature was set to 15 °C for loading the samples and warmed to 37 °C to induce gelation while measuring oscillatory moduli (storage modulus (G’) and loss modulus (G”)) at the fixed angular frequency of 1 rad/s and strain of 5%. Following gelation, a frequency sweep was performed at 37 °C, from 100 to 0.1 rad/s at fixed 5% strain; G’ and G” were measured to calculate complex viscosity for each gel. Three different batches of gels were tested from each decellularization protocol (homogenized and optimized), rat-tail collagen type I (Corning, Corning, NY) was used as a control for comparison. All gels were prepared at a concentration of 8 mg/mL. Data was collected and analyzed with Rheology Advantage software (TA Instruments, New Castle, DE), and graphs were created with Prism 6 for Windows (GraphPad Software, Inc.). Each protocol was assessed using three biological replicates, each of a different batch of gel from a different donor.

### Cell culture

Human islets were received through the Integrated Islet Distribution Program (IIDP) and experiments were initiated within 24 h of receipt. On day 0, islets were counted and plated in suspension or in hydrogel co-culture. Islets were combined with hP-HG (8 mg/mL) at a density of 100 IEQ/10μL of hydrogel. The mixture was pipetted into 5 μL droplets in the bottom of an untreated petri dish, inverted, and incubated at 37 °C and 5% CO_2_ for 30 min. The polymerized droplets were moved into 24-well ULA plates (Corning, Corning, NY) for culture for 1–7 days in PIM(R) medium (Prodo Labs, Aliso Viejo, CA), at which point they were collected for MTS, GSIS, or total insulin content. In parallel, islets were cultured in suspension in 24-well plates for the same period of time. Islets embedded in collagen 1 (8 mg/mL) (Corning, Corning, NY) followed the same protocol as hP-HG. Islets were embedded in alginate following methodology adapted from Alagpulinsa et al.^32^. Briefly, islets were mixed with 1.6% w/v sodium alginate in 150 mM NaCl, at 100 IEQ/10 μL, and manually dropped into a 100 mM CaCl_2_ bath 5 μL at a time to form droplets.

### Glucose stimulated insulin secretion and total insulin content

Static GSIS Assays were performed in series, in 24-well plates with cell filter inserts (MilliporeSigma, St. Louis, MO). Cells were added to the filters and moved from low glucose (2.8 mM) to high glucose (28 mM) to low glucose (2.8 mM) to a depolarization solution (30 mM KCl, 2.8 mM glucose). All solutions for GSIS were made in Krebs buffer (25 mM HEPES, 115 mM NaCl, 24 mM NaHCO3, 5 mM KCl, 1 mM MgCl2, 2.5 mM CaCl2, 1% BSA). The supernatant was collected following 1 h in each step of the GSIS for secreted C-pep measurement. For human islets, 100 IEQ were used per well. Supernatants collected from each treatment were frozen in aliquots. Following GSIS, cells were lysed in 1 mL of lysis buffer (20 mM Tris–HCL, pH 7.5, 150 mM NaCl, 1 mM EDTA, 1% Triton) and homogenized with a PowerGen 500 homogenizer (ThermoFisher, Waltham, MA); these lysates were used to measure total C-peptide content. C-peptide content for all lysates and supernatants were determined with an ultra-sensitive human C-pep ELISA (Mercodia, Uppsala, Sweden). Stimulation index (SI) for the static GSIS was calculated by dividing the average secreted C-peptide concentration under high glucose by the average C-peptide secreted under the first low glucose period.

Islets were prepared as described above, and assessed with perifusion GSIS on day 2 of culture. Perifusion was performed using a BioRep 4.0 semi-automated perifusion system (Biorep, Miami, FL) for 30 min of low glucose treatment (1.6 mM) followed by 45 min of high glucose treatment (16.7 mM) and return to low glucose. Flow-through samples were collected every minute from each chamber, and values from every two minutes were averaged together for graphing. For comparison, graphed values are normalized to the average low glucose response for each curve (unitless measure). The SI for the perifusion GSIS was calculated using the area under the curve (AUC) of the high glucose period per minute, divided by the AUC of the first low glucose period per minute.

### Mitochondrial respiration

Human islets were assessed for mitochondrial respiratory capacity analysis by high resolution respirometry using an Oxygraph-2k (Oroboros Instruments, Innsbruck, Austria). Islets cultured in suspension or hP-HG for 2 days, were simultaneously analyzed in two separate chambers, in 2 mL volume containing 800 IEQ each. Experiments were performed as previously described^68,69^. Briefly, mitochondrial respiration was measured by detecting mitochondrial oxygen consumption at 37 °C in standard PIM(R) medium. After establishing a basal respiration (Routine), inhibitors for the different mitochondrial respiratory complexes were added to the cells in the following order: oligomycin (2ug/ml) (MilliporeSigma, St. Louis, MO) to inhibit ATP-synthase (complex V) to measure leak respiration (Leak), carbonyl cyanide-p-trifluoromethoxyphenylhydrazone (FCCP) (MilliporeSigma, St. Louis, MO) uncoupler with step-wise titration in 0.5 to 1.5 μM increments to measure the maximal respiratory capacity of the electron transport system (ETS) (Maximal), rotenone (MilliporeSigma, St. Louis, MO) 0.5 μM final concentration to inhibit complex I, and antimycin A (MilliporeSigma, St. Louis, MO) to inhibit complex III in 2.5 μM final concentration. Data was analyzed using DatLab7 (version 7.3.0.3) (Oroboros Instruments, Innsbruck, Austria) software. The use of chambers for the 2 treatments (S and H) was switched between 3 biological replicates to avoid any possible bias due to chamber differences.

### MTS assay

100 IEQ of human islets were plated per well of 24-well ULA plates (Corning, Corning, NY). Islets were embedded in hP-HG (50 IEQ / 5 μL gel) as described above, and two 5 μL gels were placed in each well of a 24-well plate (100 IEQ/well); wells were replenished with fresh medium every 3 days. On days 1, 3, 5 and 7 after plating, the remaining cells or gels from each pre-counted well were transferred to 1.5 mL Eppendorf tubes with 300 μL of medium containing CellTiter-96 reagent (Promega, Madison, WI). The IEQ was not recounted each day, the remaining number of islets in the well were used to assess survival compared to day 0. The tubes were incubated with shaking and open caps for 3 h, at 5% CO2 and 37 °C. After incubation, the absorbance of the supernatant was measured on a spectrophotometer at 490 nm (FlexStation 3, Molecular Devices). Each treatment was tested in technical triplicate for each condition and time point.

### Histology and immunofluorescent staining

Samples were fixed in 4% paraformaldehyde (PFA), paraffin embedded, and sectioned (5 μm) for immunofluorescent staining. Slides were deparaffinized using xylene and rehydrated. Antigen retrieval was performed by incubation in 10 mM Citrate Buffer, pH 6.0 for 2.5 h at 80 °C. Slides were blocked with 10% BSA/PBS for 40 min at RT, incubated with primary antibodies overnight at 4 °C, washed, incubated with secondary antibody incubation for 40 min at RT and cover slipped. All antibodies and dilutions are listed in Supplemental Table 2. Nuclei were labeled with 40-6-diamidino-2-phenylindole (DAPI) (Life Technologies, Carlsbad, CA). Images were generated with a Zeiss Axiovert 200 M microscope or a Nikon A1R confocal microscope. TUNEL staining (ApopTag® Fluorescein In Situ Apoptosis Detection Kit) (MilliporeSigma, St. Louis, MO) was performed following manufacturer protocol, with the insulin immunostaining performed immediately afterward.

IF staining was quantified in ImageJ by tracing the Ins-positive (islet) clusters. Nuclear stains were quantified by counting the number of total nuclei (DAPI) within the islet regions and the number of TUNEL-positive and Ki67-positive nuclei in the same regions to determine the percentage of positive cells. Cellular stains were quantified by converting the images to binary and measured as percentage of islet area.

For islet architecture analysis, Ins/Gcg/Sst triple stained images were taken as z-stacks of 7 slices and quantified using the 3D Tissue Organization Toolbox plugin to determine cell type, counts and cell–cell interactions^70^. Cells were manually counted as part of the mantle if they were at the outermost edge of the islet, all other cells were counted as part of the core, as previously described^71^. To measure Tie2 and αSMA localization, whole islet clusters and islet mantles (5–10 micron width surrounding the outermost layer of islet nuclei) were manually traced in synchronized windows; positive area within each traced region was measured in all relevant channels.

For each IF analysis, 5 islet donors were assessed for each time point and condition, at least 9 islets were counted per donor and treatment.

### Quantification and statistical analysis

Data are reported as average ± standard deviation unless otherwise indicated. All p-values were calculated with a Student’s two-tailed t-test using Prism 6 for Windows (GraphPad). Prism's suggested significance classification scheme was followed (*p < 0.05), (**p < 0.01), (***p < 0.001), (****p < 0.0001).

All composite figures were prepared in Adobe Illustrator 24.0 (Adobe Inc.).

### Ethics statement

This study is reported in accordance with ARRIVE guidelines (https://arriveguidelines.org).

## Supplementary Information

Supplementary Information.

Supplementary Video 1.

## Data Availability

All data are available in the manuscript, Supplementary Information, or available from the corresponding author upon request. Raw and processed RNA sequencing data have been deposited in the NCBI Gene Expression Omnibus (GEO) repository, with the accession identifier GSE166505.

## Abbreviations

ECM: Extracellular matrix
hP-ECM: Human pancreatic extracellular matrix
hP-HG: Human pancreatic extracellular matrix hydrogel
hK-HG: Human kidney extracellular matrix hydrogel
sGAG: Sulfated glycosaminoglycan
GSIS: Glucose stimulated insulin secretion
SI: Stimulation index
IEQ: Islet equivalents
MTS: 3-(4,5-Dimethylthiazol-2-yl)-5-(3-carboxymethoxyphenyl)-2-(4-sulfophenyl)-2H-tetrazolium
SC-ILC: Stem cell-derived islet-like clusters

## Materials availability

Unique reagents generated in this study, including hP-HG and hK-HG, are available from the lead contact with a completed Materials Transfer Agreement.
