# Supplementary material for: A human pancreatic ECM hydrogel optimized for 3-D modeling of the islet microenvironment
Source: Sci Rep. 2022 May 3;12:7188. doi: 10.1038/s41598-022-11085-z (PMC9065104; doi:10.1038/s41598-022-11085-z)
Supplement: Supplementary file 1 — Supplementary Information. [file 41598_2022_11085_MOESM1_ESM.docx]

**Supplementary Information**

**Supplementary Methods**

*Cell Culture*

INS-1 832/13 were plated in a single cell suspension into 10 cm ultra-low attachment (ULA) dishes (Corning, 3262) and cultured for 3 days to form INS1 pseudoislets. On day 3, pseudoislets were counted, combined with hP-HG at a density of 200 IEQ/10μL of hydrogel and evenly distributed within the gel. The mixture was pipetted into 5 μL droplets in the bottom of an untreated petri dish, inverted, and incubated at 37 ^o^C and 5% CO_2_ for 30 minutes. The polymerized droplets were moved into 24-well ULA plates (Corning, 3473) for culture for 2 days until the GSIS was performed. For static GSIS with INS1 832/13 pseudoislets, clusters were counted using the human islet IEQ counting mechanism and 800 IEQ were used per well. (N=4 biological replicates)

*Rheology*

To assess droplet shape retention following gelation, 10 µL droplets of cold, neutralized liquid hydrogel were pipetted onto a tissue culture plate and incubated at 37 C for 30 minutes. Following gelation, droplets were imaged and droplet diameter was measured relative to the standard diameter of the tissue culture well.

Young’s Modulus was calculated using Equation 1 and Equation 2, with G’ and G” values at last time point of the time sweep (after full gelation) of the gels.

Equation 1: Complex Modulus (G)

$$G= \sqrt{{(G^{'})}^{2}+{(G'')}^{2}}$$

Equation 2: Young’s Modulus (E), with Poisson’s ratio, v = 0.5 for hydrogels.

$$E=2\times G(1+v)$$

*Bulk RNA Sequencing*

RNA was isolated from human islets after 2 days of culture in suspension or hydrogel conditions. cDNA libraries were prepared using Takara SMARTer Total RNA Seq Kit v2 Pico Input kit (Takara Bio USA, Mountain View, CA) and purified with AMPure XP Beads (Beckman Coulter, Brea, CA); RNA-Seq was performed with the Illumina NovaSeq6000 sequencing system with 200 million reads. 3 islet donors were used for each condition. Gene Ontology (GO) was performed using a list of differentially expressed genes with g:Profiler (version e99_eg46_p14_f929183) with g:SCS multiple testing correction method applying significance threshold of 0.05 ^1^.

*Transplantation*

5 µL hP-HG droplets derived using the optimized protocol and embedded with human islets (50 IEQ per 5 µL gel) were transplanted into NOD-scid IL2rγ^null^ (NSG) mice. 20 gels were transplanted within the center of a 3 mm-internal diameter silicone O-ring (Hooper, UK, OR3X1.5) in the lateral subcutaneous (SQ) space. 10 gels were transplanted into the right kidney sub-capsule (KSC) space. Images were taken at the time of surgery.

*Humanized Mice*

Research involving mice was performed in accordance with a protocol that was approved by the University of Wisconsin School of Medicine and Public Health Animal Care and Use Committee, and in accordance with a protocol approved by the University of Wisconsin Institutional Review Board. Humanized mouse models were generated using NSG mice aged 7–8 weeks, as previously described (Sackett et al., 2018). A small piece of decellularized hP-ECM was transplanted into the left dorsal subcutaneous space, and 500 μL of 10 mg/mL hP-HG was injected into the right dorsal subcutaneous space of humanized mice (N = 3 mice). Additionally, human fetal pancreas (HFP) fragments from a donor allogeneic to the donor of the mouse’s human immune system were also transplanted subcutaneously into the same animals. The three grafts remained *in vivo* for a period of four weeks before the animals were sacrificed, and grafts were collected for processing and immunohistochemistry. Upon collection of the grafts, the hP-HG graft was noticeably smaller after four weeks *in vivo*, but the large volume of the original injection and the high concentration of ECM (10 mg/mL) ensured reliable recovery of the material within this timeframe. Smaller volumes of the gel were not able to be found after this period of time *in vivo*.

*Transmission Electron Microscopy (TEM)*

Islets cultured in suspension or hP-HG for two days were fixed overnight with glutaraldehyde-based fixative (2.5% glutaraldehyde, 2.0% paraformaldehyde buffered in 0.1M sodium phosphate buffer (PB)) at 4C. The samples were rinsed 5 x 5 minutes in PB, and post-fixed in 1% osmium tetroxide, 1% potassium ferrocyanide in 0.1M PB for 1 hour at room temperature (RT), and rinsed in PB as before.

Dehydration was performed in a graded ethanol series (35, 50, 70, 80, 90% for 10 minutes each step, 95% for 20 minutes, 100% for 2 x 10 minutes) at RT and 100% EtOH at 4^o^ C overnight (ON) then transitioned in propylene oxide (PO) 2 x 7 minutes at RT. Fully dehydrated samples were infiltrated in increasing concentrations of PolyBed 812 (Polysciences Inc. Warrington, PA) and Propylene Oxide (PO) mixtures in the following order (RT mixtures with shaker table agitation):

| PolyBed 812 | PO | Time | Temp. |
| --- | --- | --- | --- |
| 10%  25% | 90%  75% | 3 hrs  ON | RT  RT |
| 50% | 50% | ON | RT |
| 75% | 25% | 2 hrs | RT |
| 100% | 0% | 45 min. | 60^o^C |

Embedding and polymerization took place in fresh PolyBed 812 for 24 hours at 60^o^C. The samples were sectioned on a Leica EM UC6 ultramicrotome at 100nm. The sections were collected on formvar coated 2x1mm slot Cu grids (EMS Hatfield, PA), and post-stained with uranyl acetate and lead citrate. The sectioned samples were viewed at 80kV on a Philips CM120 transmission electron microscope, equipped with AMT BioSprint12 digital camera (AMT Imaging Systems, Woburn, MA).

*sGAG Content*

The sulfated glycosaminoglycan (sGAG) content was quantified using Papain-digested tissue or ECM, using the Blyscan GAG Assay Kit (Biocolor, UK), following manufacturer’s protocol.

**Resources and Reagents**

| **Reagent or Resource** | **Source** | **Identifier** |
| --- | --- | --- |
| **Antibodies** | | |
| Anti-Insulin (guinea pig) | MilliporeSigma | I8510 |
| Anti-Glucagon (rabbit) | Abcam | Ab 92517 |
| Anti-Somatostatin (mouse) | Proteintech | 17512-1-AP |
| Anti-Ki67 (rabbit) | Abcam | ab16667 |
| Anti-Insulin (mouse) | MilliporeSigma | I2018 |
| Anti-Collagen 1 (rabbit) | Abcam | ab34710 |
| Anti-Collagen 4 (rabbit) | Abcam | ab6586 |
| Anti-Collagen 3 (mouse) | Abcam | ab6310 |
| Anti-Collagen 6 (rabbit) | Abcam | ab6588 |
| Anti-Laminin (rabbit) | MilliporeSigma | L9393 |
| Anti-FN1 (rabbit) | Abcam | ab2413 |
| Anti-OGN (rabbit) | Proteintech | 12755-1-AP |
| Anti-Tie2 (rabbit) | Abcam | ab221154 |
| Anti-αSMA (mouse) | MilliporeSigma | A2547 |
| Anti-CD3 (rabbit) | Abcam | ab134096 |
| Anti-CD45 (mouse) | BD Pharmingen | 555491 |
| **Chemicals and Reagents** | | |
| Sodium deoxycholate | MilliporeSigma | D6750-25G |
| PIM(R) medium | Prodo Labs | PIM-CR001GMP |
| DAPI | ThermoFisher | D3571 |
| Sodium alginate | MilliporeSigma | PHR1471-1G |
| Rat-tail Collagen 1 | Corning | 354249 |
| Pepsin | MilliporeSigma | P7012-250MG |
| HCl | MilliporeSigma | NC9894356 |
| Benzonase | MilliporeSigma | E8263 |
| Oligomycin | MilliporeSigma |  |
| FCCP | MilliporeSigma |  |
| Rotenone | MilliporeSigma |  |
| Antimycin A | MilliporeSigma |  |
| AMPure XP Beads | Beckman Coulter | A63880 |
| **Materials** | | |
| Sieve | MilliporeSigma | S1145 |
| Cell filter inserts | MilliporeSigma | PIXP01250 |
| 24-well ULA plates | Corning | 3473 |
| **Commercial Assays** | | |
| ApopTag® Fluorescein In Situ Apoptosis Detection Kit | MilliporeSigma | S7110 |
| Quant-iT™ PicoGreen® dsDNA Assay | Life Technologies | P7589 |
| Blyscan GAG Assay Kit | Biocolor | B1000 |
| Ultra-sensitive human C-pep ELISA | Mercodia | 10-1141-01 |
| SMARTer Total RNA Seq Kit v2 Pico Input kit | Takara Bio | 634411 |
| CellTiter-96 reagent | Promega | G3582 |
| **Cell Lines** | | |
| INS-1 832/13 | Hohmeier et al 2000 |  |
| **Computer Software** | | |
| Fiji | https://imagej.net/Fiji |  |
| DatLab7 (version 7.3.0.3) | Oroboros Instruments |  |
| Prism 6 for Windows | GraphPad Software |  |
| **Unique Materials** | | |
| Human pancreatic hydrogel (hP-HG) | University of Wisconsin |  |

**Supplemental Table 1: Pancreas donor information**

| **Donor** | **Sex** | **Age** | **BMI (kg/m^2^)** | **DCD/DBD** | **CIT (hrs)** |
| --- | --- | --- | --- | --- | --- |
| Donor 10 | F | 53 | 35.2 | DCD | 6 |
| Donor 23 | M | 58 | 27.4 | DBD | 6.5 |
| Donor 27 | F | 45 | 31.1 | DBD | 13.5 |
| Donor 31 | M | 21 | 27.6 | DBD | 14.5 |
| Donor 32 | M | 35 | 30.8 | DCD | 25 |
| Donor 35 | F | 48 | 20.7 | DBD | 5 |
| Donor 39 | M | 31 | 27.1 | DBD | 16 |
| Donor 40 | M | 41 | 29.3 | DBD | 8.5 |
| Donor 56 | M | 61 | 34.6 | DBD | 17 |
| Donor 65 | M | 21 | 28.8 | DBD | 7 |

Pancreas donor information. (M = male, F = female, BMI = body mass index, DCD = donation by cardiac death, DBD = donation by brain death, CIT = cold ischemia time)

**Supplemental Table 2: Immunohistochemistry information**

| **Target** | **Blocking** | **Primary** | **Secondary** |
| --- | --- | --- | --- |
|  | Blocking buffer; | antibody; host, | antibody; host, |
|  | dilution; supplier | dilution, supplier | dilution, supplier |
|  | (catalogue #) | (catalogue #) | (catalogue #) |
| Ins | Bovine Serum Albumin; 10%; Fisher Scientific (BP1600) | Insulin polyclonal; Raised in guinea pig; 1:2000; Sigma-Aldrich (I8510) | Goat anti-guinea pig A488; 1:800; Life Technologies (A11073) |
| Gcg | Bovine Serum Albumin; 10%; Fisher Scientific (BP1600) | Glucagon monoclonal; Raised in rabbit; 1:2000; abcam (ab92517) | Goat anti-rabbit A568; 1:800; Life Technologies (A11036) |
| Sst | Bovine Serum Albumin; 10%; Fisher Scientific (BP1600) | Tie2 monoclonal; Raised in rabbit; 1:4000; abcam (ab221154) | Goat anti-rabbit A568; 1:800; Life Technologies (A11036) |
| Tie2 | Bovine Serum Albumin; 10%; Fisher Scientific (BP1600) | Somatostatin monoclonal; Raised in mouse; 1:200; Proteintech (17512-1-AP) | Goat anti-mouse A647; 1:800; Life Technologies (A21235) |
| αSMA | Bovine Serum Albumin; 10%; Fisher Scientific (BP1600) | αSMA monoclonal; Raised in mouse; 1:25,000; Sigma-Aldrich (A2547) | Goat anti-mouse A647; 1:800; Life Technologies (A21235) |
| Collagen I | Bovine Serum Albumin; 10%; Fisher Scientific (BP1600) | Collagen I polyclonal; Raised in rabbit; 1:500; Abcam (ab34710) | Donkey anti-rabbit A488; 1:800; Life Technologies (A21206) |
| Collagen III | Bovine Serum Albumin; 10%; Fisher Scientific (BP1600) | Collagen III monoclonal; Raised in mouse; 1:100; Abcam (ab6310) | Donkey anti-rabbit A488; 1:800; Life Technologies (A21206) |
| Collagen IV | Bovine Serum Albumin; 10%; Fisher Scientific (BP1600) | Collagen IV polyclonal; Raised in rabbit; 1:300; Abcam (ab6586) | Donkey anti-rabbit A488; 1:800; Life Technologies (A21206) |
| Collagen V | Bovine Serum Albumin; 10%; Fisher Scientific (BP1600) | Collagen V polyclonal; Raised in rabbit; 1:100; Abcam (ab7046) | Donkey anti-rabbit A488; 1:800; Life Technologies (A21206) |
| Collagen VI | Bovine Serum Albumin; 10%; Fisher Scientific (BP1600) | Collagen VI polyclonal; Raised in rabbit; 1:100; Abcam (ab6588) | Donkey anti-rabbit A488; 1:800; Life Technologies (A21206) |
| Fibronectin | Bovine Serum Albumin; 10%; Fisher Scientific (BP1600) | Fibronectin polyclonal; Raised in rabbit; 1:200; Abcam (ab2413) | Donkey anti-rabbit A488; 1:800; Life Technologies (A21206) |
| Laminin | Bovine Serum Albumin; 10%; Fisher Scientific (BP1600) | Laminin polyclonal; Raised in rabbit; 1:200; Sigma-Aldrich (L9393) | Donkey anti-rabbit A488; 1:800; Life Technologies (A21206) |
| Mimecan (OGN) | Bovine Serum Albumin; 10%; Fisher Scientific (BP1600) | Mimecan polyclonal; Raised in rabbit; 1:50; Proteintech (12755-1-AP) | Donkey anti-rabbit A488; 1:800; Life Technologies (A21206) |
| CD45 | Bovine Serum Albumin; 10%; Fisher Scientific (BP1600) | CD45RO monoclonal; Raised in mouse; 1:2000; BD Pharmingen (555491) | ImmPRESS® HRP Horse Anti-Mouse IgG Polymer Detection Kit, Peroxidase (MP-7402); DAB substrate (SK-4105) |
| CD3 | Bovine Serum Albumin; 10%; Fisher Scientific (BP1600) | CD3G monoclonal; Raised in rabbit; 1:500; Abcam (ab134096) | ImmPRESS® HRP Goat Anti-Rabbit IgG Polymer Detection Kit, Peroxidase (MP-7451); DAB substrate (SK-4105) |

**Supplemental Table 3: Gene Expression in Islets Cultured in Suspension or hP-HG for 2 Days**

| **Symbol** | **Fold Change** | **FDR** | **Significance** | **Protein** |
| --- | --- | --- | --- | --- |
| INS | 1.261 | 0.67 | NS | insulin |
| GCG | 1.254 | 0.65 | NS | glucagon |
| SST | 1.043 | 0.94 | NS | somatostatin |
| CHGA | 0.846 | 0.63 | NS | chromogranin A |
| NKX6-1 | 1.214 | 0.23 | NS | NK6 homeobox 1 |
| UCN3 | 1.166 | 0.27 | NS | urocortin 3 |
| ARX | 0.854 | 0.26 | NS | aristaless related homeobox |

**Supplemental Video 1: Handling of hP-HG constructs**

Constructs of hP-HG and cells can be handled without damage, enabling easy transfer during culture, experimental assays, and transplantation.

**Supplemental Figure 1: Composition of the hP-ECM and hP-HG.**

**
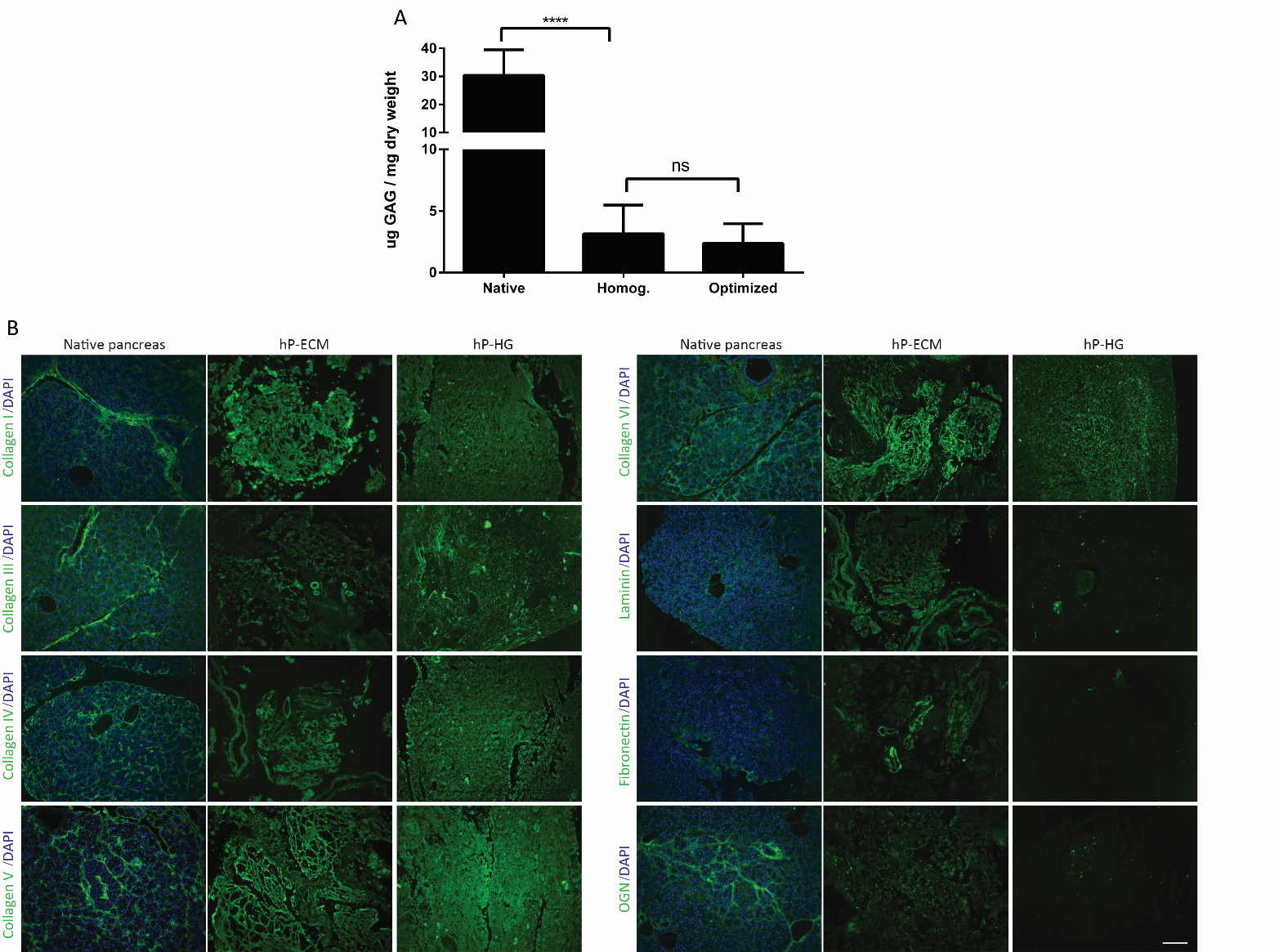
**

**Supplemental Figure 1: Composition of the hP-ECM and hP-HG.**

**(A)** Sulfated glycosaminoglycan (sGAG) content of native human pancreas compared to decellularized ECM generated with the Homog and Optimized protocols. **(B)** Immunofluorescent staining of native human pancreas, decellularized ECM and hP-HG for various ECM proteins (green), counterstained with DAPI (blue) to also indicate the removal of DNA through decellularization. Scale bar = 100 microns.


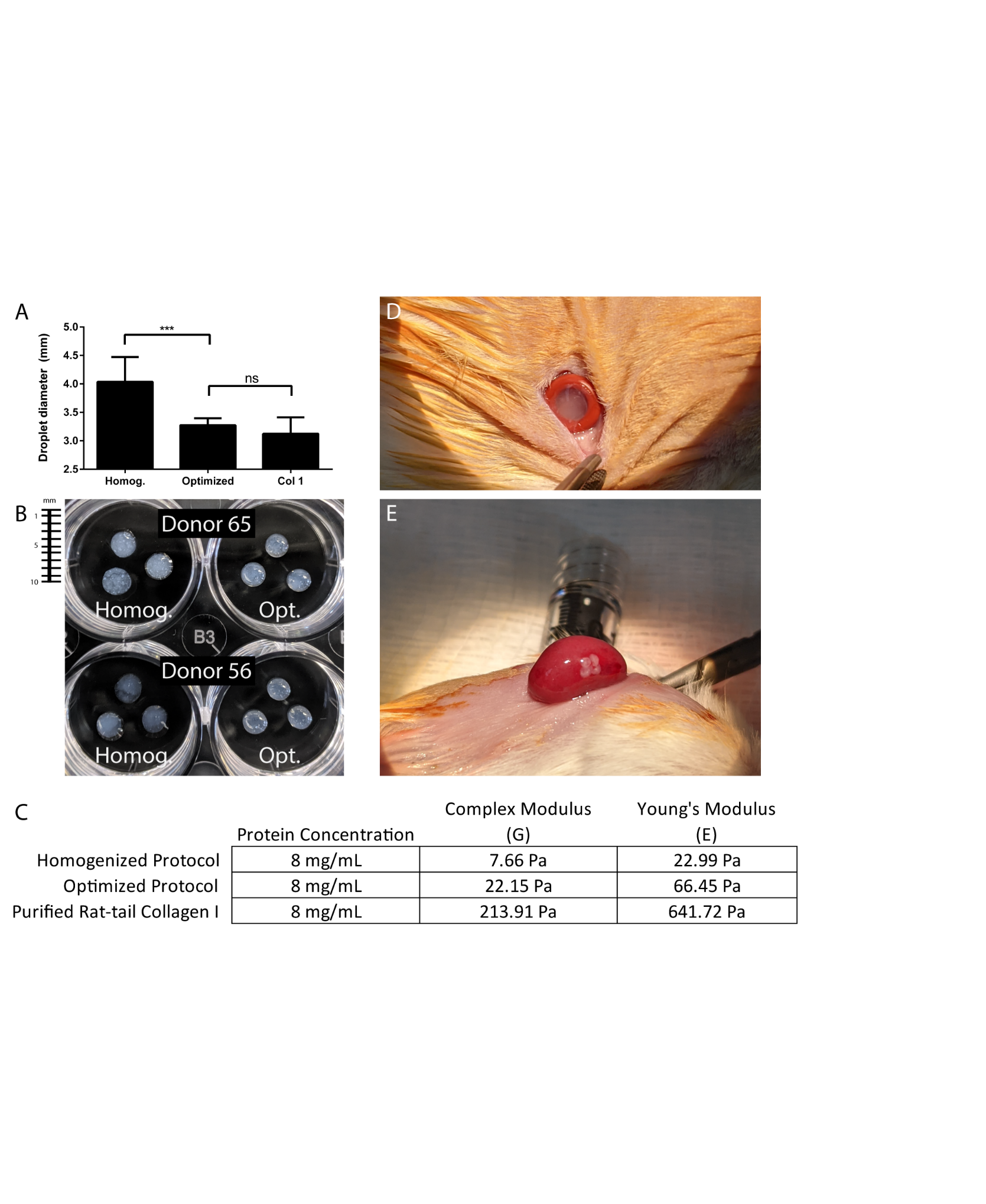


**Supplemental Figure 2: Optimized-protocol hP-HG holds shape and is transplantable**

**(A)** The diameter of 10 µL gels made from the homogenized protocol (Homog), optimized protocol (Opt) and purified Col1; N=7 donors per treatment. **(B)** Representative images of gels made from two pancreas donors, each processed with the Homog and Optimized (Opt.) protocols. For both donors, the diameters of the gels are noticeably larger following the Homog protocol compared to the Optimized protocol, indicating a lack of firmness and pliability, and a flatter gel. **(C)** Calculated Complex Modulus (G) and Young’s Modulus (E) for each measured material at 8 mg/mL protein concentration. The optimized hP-HG gels hold shape well enough that they can easily be transplanted subcutaneously (SQ) **(D)** and into the kidney sub-capsule (KSC) **(E)** without breaking or losing shape.


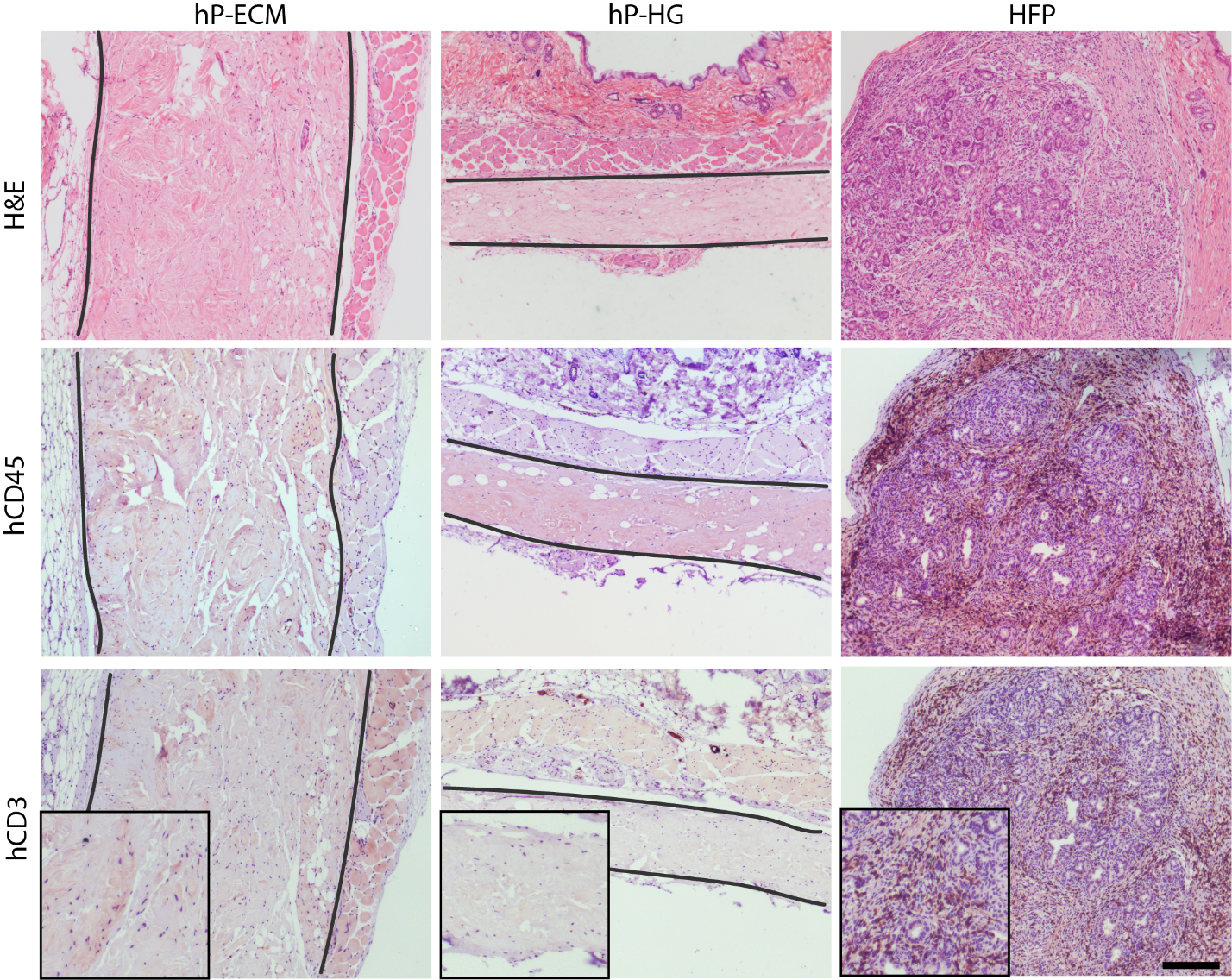


**Supplemental Figure 3: Minimal immune cell infiltration in acellular hP-ECM and hP-HG in humanized mice.**

Representative images of Optimized protocol-derived hP-ECM, hP-HG and allogeneic human fetal pancreas (HFP) grafts transplanted subcutaneously into humanized mice and collected after 4 weeks. Decellularizerd gels and ECM scaffolds were transplanted as acellular materials (without cultured cells) to test immunogenicity of the scaffolds themselves. Each mouse was transplanted with all three materials (N=3 mice). Grafts were stained with H&E to examine general immune infiltration, human CD45 (hCD45) to assess infiltration of human immune cells, and human CD3 (hCD3) to assess infiltration of cytotoxic T cells. hP-ECM and hP-HG had extremely low levels of any human immune cell infiltration, while allogeneic HFP in the same animal was heavily infiltrated with both hCD45+ and hCD3+ cells. Insets show more closely the positive (brown) or negative (only purple nucleus) staining for human CD3 within the different explants. Scale bar = 200 microns.


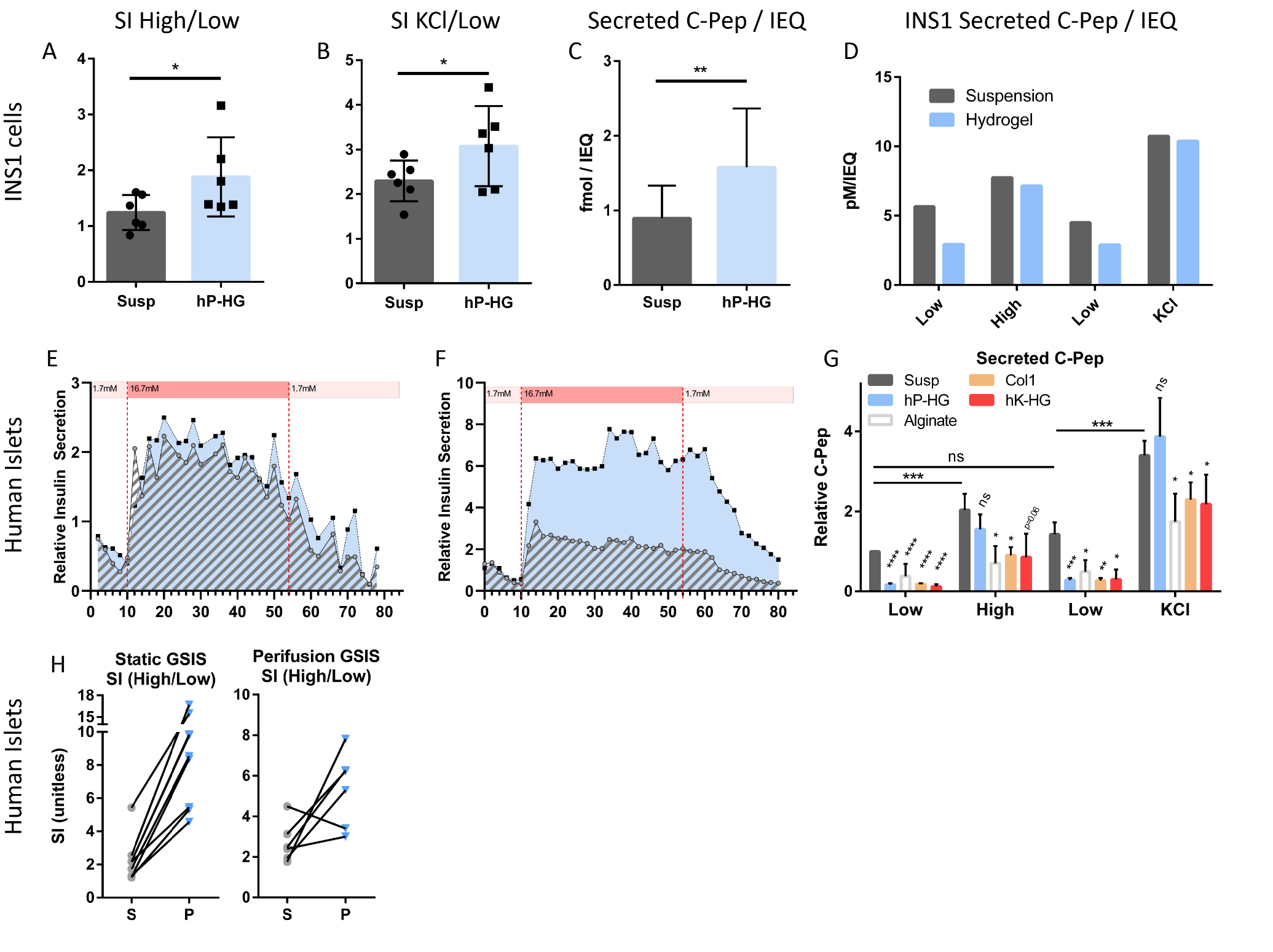


**Supplemental Figure 4: Additional GSIS data after 2 days of culture.**

Stimulation index **(A**, high/low glucose**)** **(B**, KCl/low glucose**)** of INS1 cells cultured as pseudo-islets in suspension or hP-HG, after 2 days of culture, determined by static GSIS. **(C)** Total C-Pep content, and **(D)** Insulin secretion (pM per IEQ) from INS1 cells cultured in suspension (gray) and hP-HG (blue) and exposed to low glucose (2.8 mM), high glucose (28 mM), low glucose and then KCl (30 mM). N=4 biological replicates. **(E-F)** Perifusion GSIS curves for two additional human islet donors; insulin secretion is normalized to first low glucose response (unitless). **(G)** Human islet static GSIS data presented with an alternative normalization, where all measurements for each islet donor and condition are normalized to the C-Pep level secreted under the first low glucose step. This method of analysis reveals that when not normalized as a percentage of total C-Pep content (as in Fig. 3B) the stimulated C-Pep secretion levels (High and KCl) of A,C, and K are significantly lower than those of S and P. **(H)** Stimulation index (high/low) of islets in suspension (S) or hP-HG (P) under static or perifusion GSIS, tracing the SI between islets from the same donor in each treatment group.


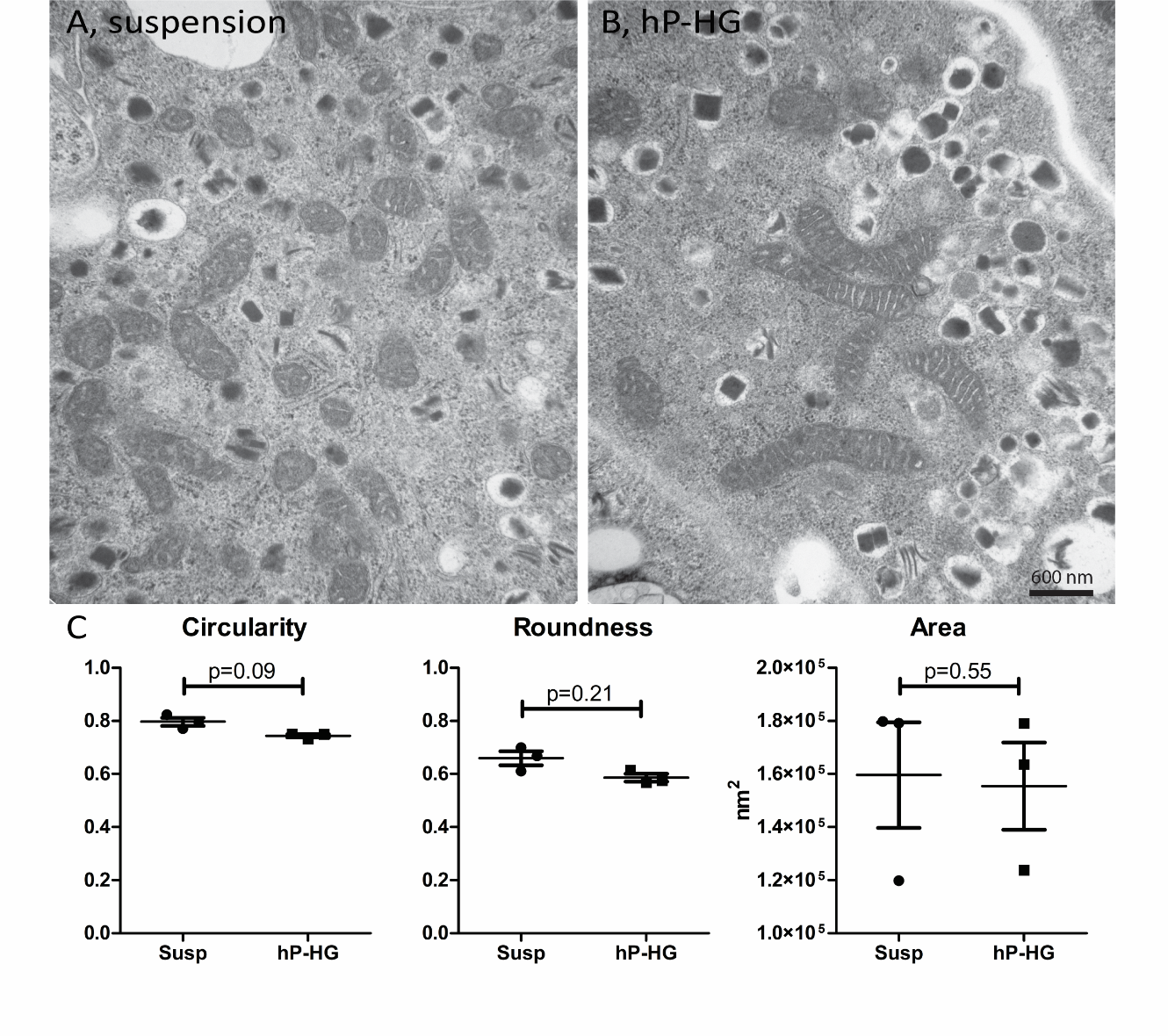
**Supplemental Figure 5: Human islet mitochondria quantifiable phenotypes**

TEM images taken at 25000x were used to quantify mitochondrial phenotypes in islets cultured in suspension **(A)** or hP-HG **(B)** for two days. N=3 islet donors. Scale = 600 nm. **(C)** The circularity (unitless), roundness (unitless), and area (nm^2^) of individual mitochondria from islets cultured in suspension or hP-HG for two days were found to not be statistically different. 98-163 mitochondria were measured per donor for each treatment, N=3 donors.


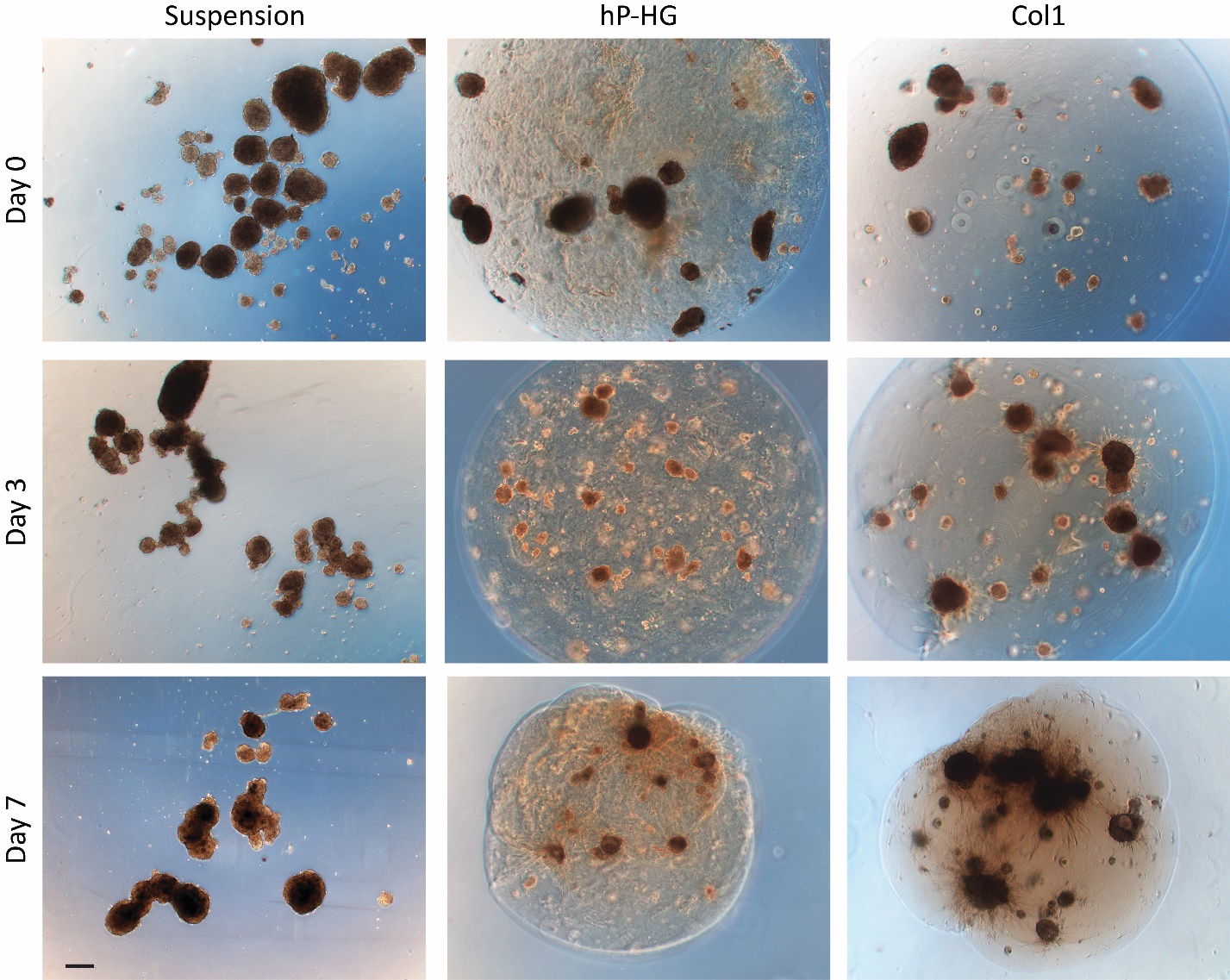


**Supplemental Figure 6: Human islet survival and growth over 7 days**

Representative bright field images of human islets cultured in suspension (left column), hP-HG (middle column), and Col1 hydrogel (right column) on day 0, the day the islets were embedded, 3 days after embedding, and 7 days after embedding. Scale = 200 microns.

**Supplemental Figure 7: Human islet architecture**


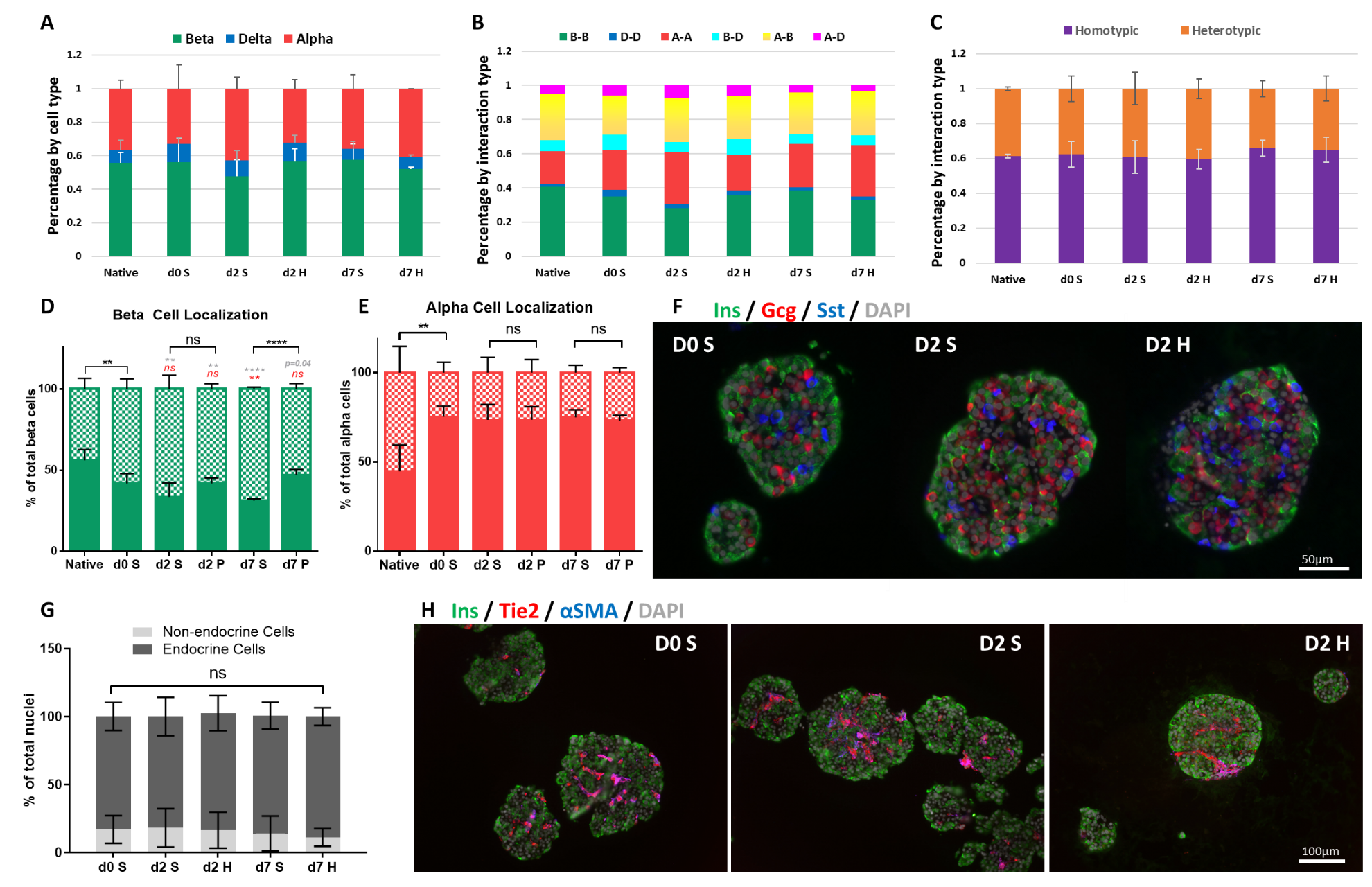


**Supplemental Figure 7: Human islet architecture**

Quantification of human islet architecture measuring the total numbers of alpha, beta and delta cells **(A)**, and the interactions between endocrine cell types among the 5 conditions **(B)** and simplified into homotypic and heterotypic interactions **(C)**. Quantification of the core:mantle arrangement of beta **(D)** and alpha **(E)** cells as a percentage of the total same cell type (core=filled bars, mantle=checkered bars). N=5 islet donors. Statistical comparisons indicated in italics and gray are compared to native islets and in italics and red are compared to “Day 0” islets. (ns = not significant, * p<0.05, ** p<0.01, *** p<0.001, **** p<0.0001) **(F)** Representative images of isolated islets in day 0 and day 2 in suspension (S) or hydrogel (H) culture, stained for insulin (green), glucagon (red), somatostatin (blue) and DAPI (gray), scale = 50 microns. **(G)** Counts for endocrine (dark gray) and non-endocrine (light gray) cells under each condition and time point, as a percentage of total nuclei counted. **(H)** Representative images of isolated islets in day 0 and day 2 in suspension (S) or hydrogel (H) culture, stained for insulin (green), Tie2 (red), αSMA (blue) and DAPI (gray), scale = 100 microns.


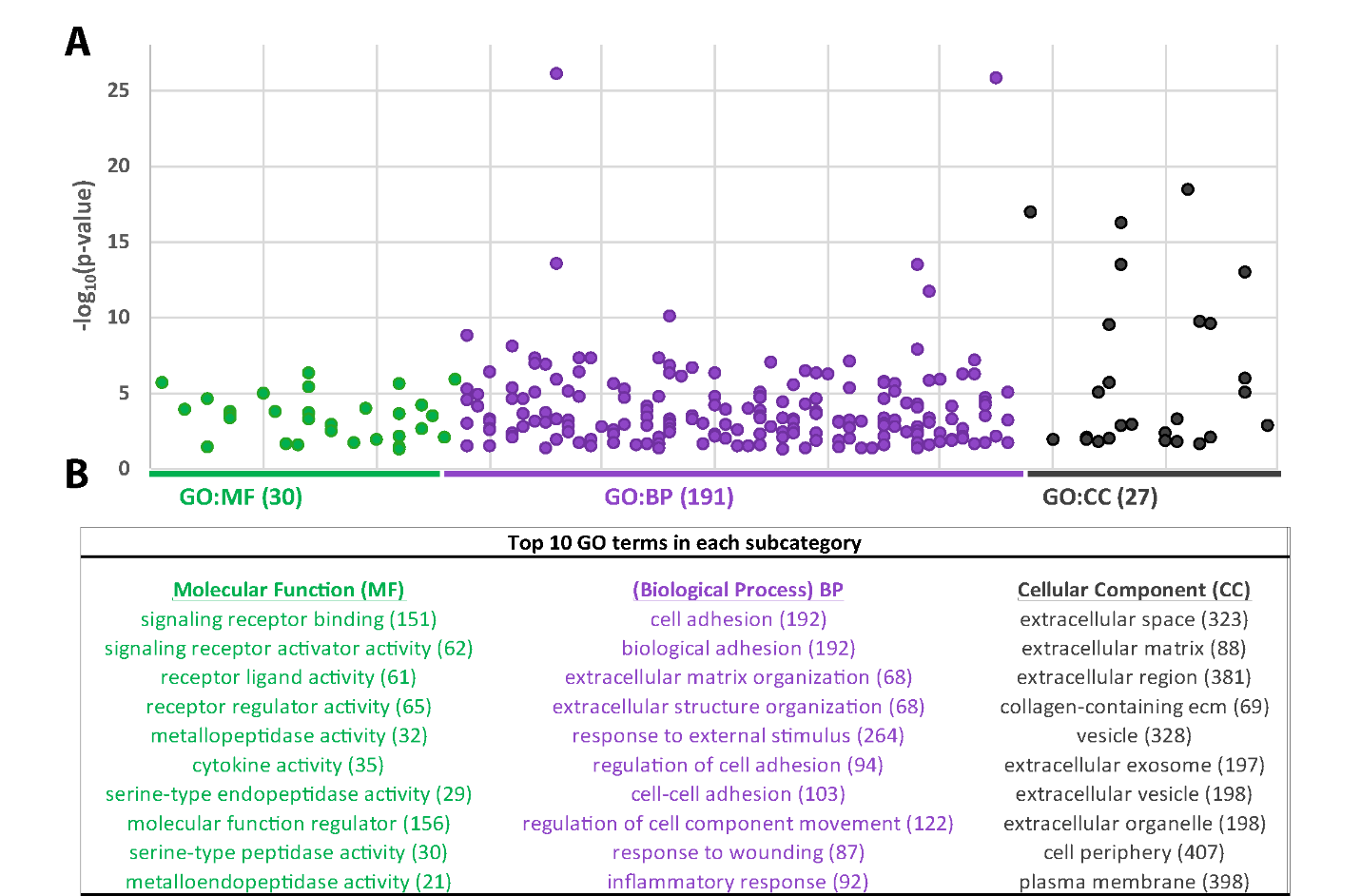


**Supplemental Figure 8: Extracellular signaling-related pathways are significantly influenced by hP-HG culture**

**(A)** Plots showing the three aspects of GO analysis: Molecular Function (MF), Biological Process (BP) and Cellular Component (CC). All pathways found to be significantly altered between suspension and hP-HG treatments are plotted, with the -log_10_(p-value) representing significance on the y-axis. Total number of gene sets identified in each aspect are in parentheses. (N=3 islets donors, each used for both treatments)

**(B)** List of the top 10 terms identified in each aspect, with the number of altered differentially expressed genes (DEGs) in that pathway in parentheses to the right.

**Supplemental References:**

1. Raudvere, U.*, et al.* g:Profiler: a web server for functional enrichment analysis and conversions of gene lists (2019 update). *Nucleic Acids Res* **47**, W191-W198 (2019).
